# Comparing human vs. machine-assisted analysis to develop a new approach for Big Qualitative Data Analysis

**DOI:** 10.1371/journal.pdig.0000576

**Published:** 2026-02-25

**Authors:** Sam Martin, Emma Beecham, Emira Kursumovic, Richard A. Armstrong, Tim M. Cook, Noémie Déom, Andrew D. Kane, Sophie Moniz, Jasmeet Soar, Cecilia Vindrola-Padros, Collaborators

**Affiliations:** 1 Oxford Vaccine Group, Department of Paediatrics, Oxford University, Oxford, United Kingdom; 2 Department of Targeted Intervention, Rapid Research Evaluation and Appraisal Lab (RREAL), University College London (UCL), London, United Kingdom; 3 Department of Anaesthesia, Royal United Hospitals Bath NHS Foundation Trust, Bath, United Kingdom; 4 Health Services Research Centre, Royal College of Anaesthetists, London, United Kingdom; 5 Department of Anaesthesia, Severn Deanery, Bristol, United Kingdom; 6 Department of Anaesthesia, Royal United Hospital, Bath, United Kingdom; 7 University of Bristol School of Medicine, Bristol, United Kingdom; 8 Medical School, University College London (UCL), London, United Kingdom; 9 Department of Anaesthesia, James Cook University Hospital, South Tees NHS Foundation Trust, Middlesbrough, United Kingdom; 10 Department of Anaesthesia, Southmead Hospital, North Bristol NHS Trust, Bristol, United Kingdom; Australian Artificial Intelligence Institute, University of Technology, AUSTRALIA

## Abstract

**Background:**

The exponential growth of Big Qualitative (Big Qual) data in healthcare research presents methodological challenges for traditional analysis approaches. This study evaluates the effectiveness of machine-assisted analysis using artificial intelligence (AI) tools compared to human-only analysis for processing large-scale qualitative datasets, using the Royal College of Anaesthetists’ 7th National Audit Project (NAP7) baseline survey as a test case.

**Methodology/Principal Findings:**

We conducted a comparative methodological study analysing 5,196 free-text responses about peri-operative cardiac arrest experiences. Three researchers established a human-coded reference standard following SRQR guidelines. We then applied machine-assisted analysis using Pulsar for exploratory analysis and Caplena for sentiment and thematic analysis, evaluating performance against the human gold standard using STARD-AI reporting standards. Performance metrics included accuracy, precision, recall, F1-scores, and Cohen’s Kappa, with confidence intervals calculated using bootstrap resampling.

Machine-assisted analysis substantially reduced analysis time, with particularly dramatic improvements in theme identification speed. The machine-assisted approach achieved good thematic and sentiment classification accuracy compared to the human reference standard, though human analysis identified an emergent ‘ambiguous’ sentiment category that current AI tools cannot accommodate, highlighting limitations in commercial platforms’ flexibility for inductive analysis.

**Conclusions/Significance:**

Machine-assisted analysis offers substantial efficiency gains with acceptable accuracy trade-offs for large-scale qualitative data analysis. However, human expertise remains essential for capturing nuanced meanings, identifying emergent categories, and providing domain-specific interpretation. This hybrid approach represents a viable methodology for Big Qual research, though current AI tools’ constraints in accommodating emergent classification schemes remain a limitation. Our findings establish benchmarks for future development of more flexible AI systems adapted to qualitative research paradigms.

## Author summary

The use of Artificial intelligence (AI) in health research has grown over recent years. However, analysis of large qualitative datasets known as Big Qualitative Data, in public health using AI, is a relatively new area of research. Here, we use novel techniques of human-machine learning known as ‘machine-assisted’ analysis and natural language processing where computers learn how to handle and interpret human language, to analyse a large national survey. The Royal College of Anaesthetists’ 7th National Audit Project is a large UK-wide initiative examining peri-operative cardiac arrest. We use the free-text data from this survey to test and validate our novel methods and compare analysing the data by hand (human) vs. machine-assisted analysis. Using two AI tools to conduct the analysis we found that the machine-assisted analysis significantly reduced the time to analyse the dataset. Extra human input, however, was required to provide topic expertise and nuance to the analysis. The AI tools reduced the sentiment analysis to positive, negative or neutral, but the human input was able to identify a fourth ‘ambiguous’ category. The insights gained from this approach present ways that AI can help inform targeted interventions and quality improvement initiatives to enhance patient safety, in this case, in peri-operative cardiac arrest management.

## Introduction

The exponential growth of qualitative data in health research presents both unprecedented opportunities and significant methodological challenges. Big Qualitative (Big Qual) data, defined as datasets containing at least 100 participants either as a stand-alone project or within a large quantitative study [1], has become increasingly common in healthcare research. Large-scale surveys, patient feedback systems, and digital health platforms now routinely generate thousands of free-text responses that require analysis. However, traditional qualitative analysis methods, whilst rigorous and nuanced, struggle to efficiently process such volumes of data without substantial time and resource investment.

This methodological challenge has prompted researchers to explore computational approaches to qualitative data analysis. Natural language processing (NLP) and machine learning (ML) techniques offer promising solutions for handling large-scale textual data [2]. Recent advances in generative AI and transformer-based models have revolutionised text processing capabilities, enabling more sophisticated understanding of context, sentiment, and thematic content than ever before [3–5]. However, the application of these technologies to qualitative health research remains in its early stages, with the community still establishing appropriate methods, standards, and tools for Big Qual data analysis [6].

Whilst AI-assisted analysis offers efficiency advantages, critics rightly note that purely automated approaches may miss the nuances and contextual understanding that human qualitative analysis provides [7]. Human analysis brings interpretive depth, domain expertise, and the ability to recognise subtle patterns and meanings that current AI systems may overlook [8,9]. Conversely, human-only analysis faces challenges of fatigue, inter-rater variability, and practical limitations when dealing with thousands of responses [10]. This has led to proposals for hybrid “machine-assisted” approaches that combine the efficiency of AI with the interpretive expertise of human researchers [11].

The integration of machine-assisted methods into qualitative research also raises important epistemological questions about the nature of knowledge production in qualitative inquiry. Traditional qualitative research often operates within interpretivist or constructivist paradigms that emphasise meaning-making and contextual understanding. The introduction of computational methods requires researchers to consider how these tools align or potentially conflict with established qualitative methodologies and what constitutes valid knowledge when machines participate in the analytical process.

To explore these methodological questions, we present a comparative analysis of human versus machine-assisted approaches using data from the Royal College of Anaesthetists’ 7th National Audit Project (NAP7) as a case study. NAP7 investigated peri-operative cardiac arrest across the UK, generating over 10,000 survey responses from anaesthetists about their experiences, practices, and perspectives [12,13]. This dataset provides an ideal test case for comparing analytical approaches due to its size, complexity, and the critical nature of the patient safety issues it addresses.

This study adopts a ‘small q’ qualitative approach, analysing structured free-text survey responses rather than the ‘big Q’ approaches typical of traditional qualitative research (such as in-depth interviews or ethnographic observation). Whilst this positions our work at the intersection of quantitative and qualitative methodologies, it offers several advantages for testing AI-assisted analysis methods: standardised question prompts allow for systematic comparison between human and machine coding; large sample sizes enable robust statistical evaluation; and the bounded nature of survey responses provides a controlled environment for assessing AI performance. We acknowledge that our findings may not fully transfer to more unstructured qualitative data such as interview transcripts or field notes, where context, researcher reflexivity, and interpretative depth play larger roles. Nevertheless, the proliferation of free-text survey data in healthcare research makes this an important domain for developing and validating machine-assisted analysis methods.

Therefore, this study has three primary objectives:

1. To compare the effectiveness of machine-assisted versus human-only qualitative analysis in identifying themes and sentiments in healthcare free-text data
2. To evaluate the efficiency gains (time and resource utilisation) of machine-assisted analysis approaches
3. To assess the limitations of current AI tools and identify where human expertise remains essential for nuanced interpretation

We test these objectives using free-text responses from the Royal College of Anaesthetists’ 7th National Audit Project (NAP7) baseline survey on perioperative cardiac arrest experiences (see S2 Appendix for NAP7 baseline survey).

## Methodology

### Human-only analysis process

Themes were developed using a hybrid inductive-deductive approach that balanced theoretical frameworks with empirical discovery. The deductive element drew upon the NAP7 survey question structure and established domains from the patient safety literature, including teamwork, communication, guidelines, and debriefing practices. This provided an initial analytical framework grounded in existing knowledge. However, we remained open to emergent patterns, allowing sub-themes and the ‘ambiguous’ sentiment category to develop inductively through iterative review of the data. This hybrid approach enabled us to maintain theoretical coherence whilst capturing unexpected nuances in the responses.

Three researchers (SM1, SM2, ND) independently reviewed the complete dataset of 5,196 responses. For thematic coding, researchers assigned one or multiple theme labels to each response, drawing from a pre-defined codebook that evolved throughout the analysis. Sentiment coding involved assigning labels based on the overall tone of each response, initially using three categories (positive, negative, neutral) which were later expanded to include ‘ambiguous’ as patterns emerged during coding. The coding process took place over 6 months, with regular team meetings held fortnightly to discuss ambiguous cases, resolve disagreements, and refine the coding framework iteratively.

Several measures ensured the trustworthiness and rigour of the human analysis. Credibility was enhanced through the involvement of multiple coders from diverse professional backgrounds: a digital health researcher, an anaesthetist, and a research assistant. This diversity of perspectives reduced the risk of discipline-specific blind spots and enriched the interpretation of responses. Dependability was maintained through the use of a structured coding framework and regular calibration meetings, which ensured consistency in how codes were applied across the dataset. Confirmability was achieved by documenting all coding decisions and maintaining a comprehensive audit trail, allowing the analytical process to be traced and verified. The inter-coder reliability, measured using Cohen’s Kappa (κ = 0.82), indicated substantial agreement between analysts, demonstrating that the human-coded dataset constituted a reliable reference standard against which to evaluate machine-assisted analysis.

Our methodological approach adopts a critical realist position [14]: we acknowledge that respondents’ survey answers reflect their genuine experiences and perceptions (a ‘real’ substrate), but we also recognise that interpretation of these experiences - whether by humans or machines - involves subjective judgement and is influenced by the interpreter’s perspective and framework. We do not claim that a single ‘true’ coding exists for each response; rather, we treat the consensus human coding as a practical reference standard that represents expert judgement informed by domain knowledge, contextual understanding, and reflexive interpretation. This reference standard allows us to evaluate machine-assisted performance whilst acknowledging that both human and machine interpretations are constructs rather than absolute truths. The value of the comparison lies in understanding where AI approximates expert judgement and where it diverges in ways that matter for research quality and clinical insight.

### Analytical framework and rationale

We selected three complementary analytical approaches, each addressing different research needs:

1. Sentiment analysis: To systematically capture the emotional valence of anaesthetists’ experiences and identify where support or intervention may be most needed. Sentiment analysis is particularly valuable in large datasets where manual assessment of emotional tone would be resource-intensive. This approach aligns with patient safety research’s increasing recognition that healthcare professionals’ emotional responses to adverse events significantly impact wellbeing, learning, and future practice.
2. Thematic analysis: To identify the substantive content and recurring topics within responses. This allows us to understand what anaesthetists are experiencing and talking about (e.g., teamwork, guidelines, debriefing), complementing sentiment analysis which tells us how they feel about these topics.
3. Discourse analysis: To explore the relationships between themes and examine how different concepts co-occur and interact within responses. This analytical layer helps identify patterns such as when poor teamwork associates with negative outcomes, or when effective leadership coincides with positive experiences despite challenging circumstances.

Together, these three analytical approaches provide a comprehensive understanding of both the content and emotional dimensions of anaesthetists’ experiences with perioperative cardiac arrest.

### Data preparation

The primary aim of this study was to compare the effectiveness and efficiency of machine-assisted analysis, using the AI tools Infranodus v5, 2023 (Nodus Labs, Ways Ltd, Leeds, UK), an AI-powered text analysis and visualisation tool which uses network analysis and knowledge graphs [15], and Caplena v2 (Caplena AG, Zurich, Switzerland) an AI-powered thematic and sentiment analysis platform which uses advanced NLP and ML techniques, against human-only analysis in the context of qualitative data from the NAP7 Baseline Survey.

By employing a comparative approach, we sought to assess the potential benefits and limitations of integrating AI tools into the qualitative research process, while maintaining the nuance and context provided by human researchers. This comparative methodology was applied throughout the data preparation, analysis, and interpretation stages of the study. First, all 10,746 responses were downloaded from the SurveyMonkey survey (Niskayuna, NY: Momentive Inc.) to Excel. Two researchers, one digital analyst and one anaesthetist (SM1, EK), cleaned the data in Excel over two months. This process involved checking responses, creating a coding key to explain medical terms/slang for non-medical team members, adapting response categories, removing duplicates, and converting free-text responses for each question into a CSV file. Through an iterative process of checking themes and sub-themes from the free-text responses - researchers (EK and SM1) chose questions to analyse based on their relevance to the impact of peri-operative events on anaesthetists’ well-being and confidence. They chose six main questions (Q2, Q4, Q30, Q36, Q41 and Q43) that best answered the related themes and quantitative findings of the main paper ^12^ - giving a final sample of 5,196 responses see S3 Appendix for a Flowchart representing the study sample. These included confidence levels in managing peri-operative cardiac arrest, insights into debriefing practices, having a rest break, or stopping work after being involved in a cardiac arrest, the prevalence of informal vs. formal well-being support and improvements needed in the post-cardiac arrest management.

### Exploratory analysis with Pulsar

Next, quantitative free-text analysis was performed using machine-assisted methods via the Pulsar Platform v2022 (Pulsar TRAC, first-party data tool, Pulsar Platform, London, UK). Pulsar is a comprehensive text analytics and social listening platform that combines natural language processing with data visualisation tools. For this study, we utilised Pulsar’s text mining capabilities to conduct initial exploratory analysis of the dataset.

Specifically, Pulsar performed segmentation analysis to divide responses by demographic factors (gender, career stage, clinical role), enabling us to identify whether particular subgroups expressed distinct patterns of experience or concern. Keyword extraction and frequency analysis identified the most commonly mentioned terms and concepts across the dataset, providing a data-driven overview of salient topics. Pulsar’s topic clustering algorithms generated an initial thematic map by identifying groups of responses with similar linguistic patterns and content. These capabilities allowed us to systematically explore the dataset’s structure and identify preliminary themes before conducting more detailed analysis.

The outputs from Pulsar—including keyword frequencies, demographic patterns, and initial topic clusters—provided a quantitative foundation for the subsequent in-depth thematic and sentiment analysis using Caplena and human review. This multi-stage approach ensured that our detailed qualitative analysis was informed by systematic exploration of the dataset’s overall structure and patterns.

We consulted the Standards for Reporting Qualitative Research (SRQR) guidelines to present the methodological process and the results [16]. Additionally, for the AI-assisted component of this study, we followed the Standards for Reporting Diagnostic Accuracy Studies - Artificial Intelligence (STARD-AI) guidelines to ensure transparent reporting of the AI system’s development, validation, and performance [17]. This dual framework approach reflects the hybrid nature of our study, which establishes human annotation as the reference standard (reported via SRQR) and then evaluates machine-assisted performance against this standard (reported via STARD-AI). Following STARD-AI reporting standards, below we describe:

Reference standard (human annotation); Three independent researchers (SM1, SM2, ND) manually coded all 5,196 responses; Coding was performed blind to machine outputs; Inter-annotator agreement (Cohen’s κ = 0.82) established reliability; Discrepancies resolved through consensus discussion; The human-coded dataset served as the reference standard for machine-assisted evaluation.

### Sentiment analysis

Sentiment analysis was conducted to systematically capture the emotional valence of anaesthetists’ experiences. Initially, we employed a three-category framework aligned with the capabilities of the AI tools: positive (responses expressing favourable experiences, satisfaction, or affirmative sentiments), negative (responses indicating dissatisfaction, concern, or adverse experiences), and neutral (objective, factual statements without clear emotional valence). However, during the human annotation process, researchers identified a recurring pattern of responses that did not fit neatly into these predefined categories. This led to the inductive development of a fourth category through iterative review of the data, consistent with grounded theory principles where categories emerge organically from the coding process. The ‘ambiguous’ category was defined to capture responses expressing mixed sentiments (containing simultaneous positive and negative elements) or where the overall sentiment was genuinely unclear despite containing emotional content. This category differed meaningfully from ‘neutral’ classifications. Neutral statements were largely factual and unemotional, whereas ambiguous statements contained conflicting or nuanced emotions that could not be reduced to a single sentiment. For example, the response “The team handled the situation well, but the lack of clear protocols made it very stressful” contains both positive elements (effective teamwork) and negative elements (protocol gaps, stress), creating genuine ambiguity in the overall sentiment. Similarly, “Good initial outcome. Although very stressful as we were underprepared and under-resourced. Felt isolated as no team support immediately after the event” progresses from positive to negative sentiment within a single response, making binary classification inappropriate. The emergence of the ‘ambiguous’ category represented a significant finding in itself: it highlighted limitations in current AI sentiment analysis tools, which were constrained to the original three-category framework and could not identify or classify ambiguous sentiments. This technical constraint is discussed as a key finding regarding the capabilities and limitations of commercial AI tools for healthcare qualitative analysis.

### AI model description and training parameters

#### Model description.

In our study, we used Caplena to conduct sentiment and thematic analysis of qualitative survey data. Caplena employs a Transformer-based architecture with a heavily modified model, leveraging multi-label classification to assign zero, one, or multiple topics per response [18,19].

The AI model underwent a three-step training process:

1. Pretraining: The model was initially trained on a large corpus of unstructured text data (>300M text comments from survey, social media and internet data), allowing it to develop a foundational understanding of language structures, including synonyms, antonyms, and negations.
2. Supervised Training on Caplena’s Data: The model was refined using millions of hand-labelled text responses (~10M annotated rows) from various domains.

Fine-Tuning on Project-Specific Data: Human reviewers manually adjusted topic and sentiment assignments on a subset (~10%) of our dataset, allowing the AI to adapt its classification for greater domain-specific accuracy. This iterative correction method, known as active learning, improved the model’s ability to correctly identify themes and sentiments in the dataset.

#### Training parameters [20,21].

- Tokenisation: Uses subword tokenisation for handling diverse terminology (this breaks text into smaller units (tokens) for computer processing and allows the AI to understand words and phrases in context, as well as better handle specialized medical terminology).
- Embedding Size: 512-dimensional vectors for input text encoding (this provides enough complexity to capture subtle meaning differences and helps distinguish between clinical terms with similar meanings but different implications).
- Attention Mechanism: Multi-headed self-attention, enabling context-sensitive text analysis (this allows the AI to focus on important words in a sentence when making decisions).
- Fine-Tuning Method: Data-driven correction via “Focus Mode,” which prioritises ambiguous cases for human review.

#### Model evaluation and performance metrics.

We employed multiple complementary metrics to evaluate the machine-assisted approach: accuracy, precision, recall, F1-score, Cohen’s Kappa (κ) for inter-annotator agreement, time efficiency, and processing capacity. Detailed definitions of these metrics are provided in S4 Appendix for readers unfamiliar with classification evaluation. Cohen’s Kappa was calculated for both human-human agreement (to establish reliability of the reference standard) and human-machine agreement (to assess AI performance relative to expert human coding). Time efficiency measured the total time required to complete the analysis, broken down by specific tasks (data preparation, coding, verification). Processing capacity was calculated as the number of responses that could be analysed per hour, determined by dividing the total number of responses by the total analysis time. All performance metrics are reported with 95% confidence intervals calculated using stratified bootstrap resampling with 1,000 iterations to account for variability in classification performance.

### Data analysis and statistical methods

All data analysis was conducted using Python (version 3.9) with the scikit-learn library (version 1.2.0) for performance metric calculations. For multi-label classifications in the thematic analysis, we calculated metrics using micro-averaging, which treats all theme assignments across all responses as a combined binary classification problem. This approach was appropriate given that responses could be assigned multiple themes simultaneously. For sentiment analysis, which employed single-label classification, we used standard multi-class classification metrics.

Performance metrics including accuracy, precision, recall, and F1-scores were calculated by comparing machine-generated classifications against the consensus human-coded reference standard. To quantify uncertainty in these estimates, 95% confidence intervals were calculated using stratified bootstrap resampling with 1,000 iterations. This method randomly sampled (with replacement) from the dataset, recalculated metrics for each sample, and derived confidence intervals from the distribution of bootstrap estimates.

Inter-annotator agreement was assessed using Cohen’s Kappa coefficient. For human-human agreement, we calculated Kappa pairwise between all combinations of human annotators and then averaged the results to obtain an overall measure of human coding reliability. For human-machine agreement, the consensus human code served as the reference standard, and the machine-generated code served as the comparator, with Kappa calculated to quantify the level of agreement.

Time and efficiency analyses were based on prospectively logged time data. All researchers recorded time spent on each stage of the analysis process (data preparation, coding, verification, refinement) using structured time-tracking logs. Processing capacity was calculated as the number of responses analysed divided by the total time invested, expressed as responses per hour. Cost estimates were based on standard UK research assistant hourly rates to enable comparison of resource requirements between approaches.

### Evaluation process

The evaluation process followed these steps: First, three researchers (SM1, SM2, ND) manually coded all 5,196 responses, assigning themes and sentiment labels. This process was timed to establish baseline efficiency metrics. Second, we calculated inter-annotator agreement rates and Cohen’s Kappa between the human annotators to establish the reliability of the human gold standard. Third, we processed the same dataset using the Caplena and Infranodus tools, with human verification and refinement of approximately 10% of the results. This process was also timed to compare with the human-only approach. Fourth, we directly compared the outputs of both approaches, calculating all performance metrics and identifying areas of agreement and disagreement. Finally, we conducted qualitative error analysis of cases where the machine and human analyses diverged to identify patterns and potential areas for improvement.

#### Data analysis – Natural language processing.

Natural language processing (NLP) analytics were combined with human input for sentiment analysis. Using Caplena, each response or statement was assigned a topic/theme label or sub-topic/theme. This meant that a response might have three sub-topics/themes attached to it. To train the Caplena model, five researchers including social scientists, a digital analyst, and an anaesthetist (EB, SM2, ND, SM1, EK,) reviewed a small subset of around 10% of the responses for each question, adding themes and sub-themes, revising labelling, and at times correcting labels. The model then learned from the changes made by the researchers and adjusted its labelling of the dataset to improve accuracy [20,22]. Researchers documented the changes they made in each section before any subsequent changes to evaluate how well the model learned from human coding and to ensure consistency.

#### Data analysis – Sentiment analysis.

**Fig 1:**
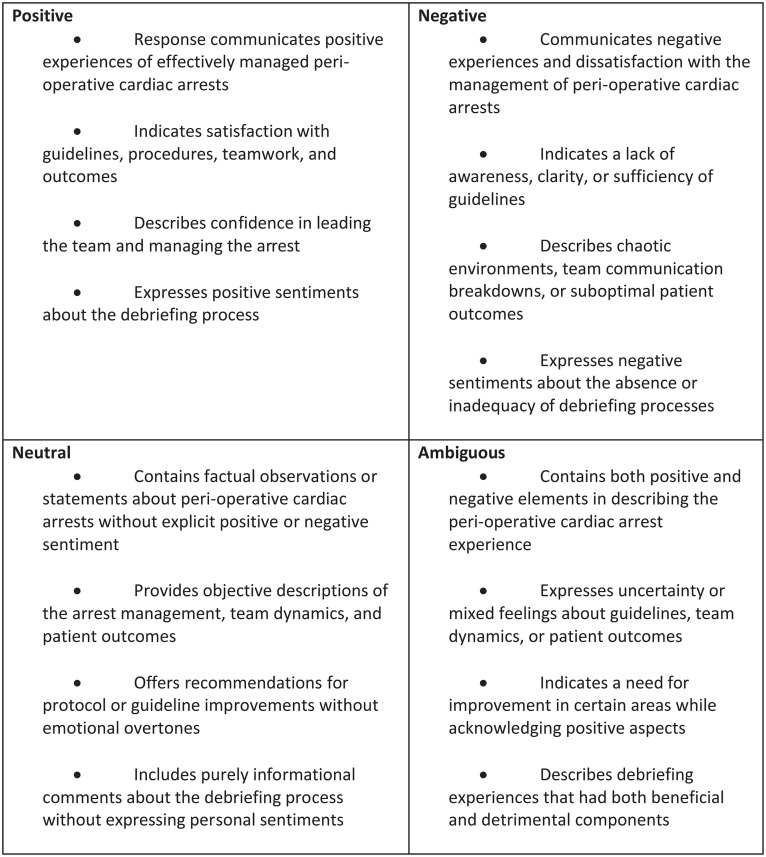
Sentiment analysis framework: Definitions of positive, negative, neutral and ambiguous sentiment – coded by human annotators.

A sentiment analysis framework was established to categorise sentiments in the Baseline Survey responses (Fig 1). This framework guided the training of sentiment and thematic analysis tools. Three researchers (EB, SM1, EK) were involved in the initial manual annotation of the data, adding columns for the overarching sentiments: positive, negative, and neutral.

Additionally, to ensure clarity and consistency in our analysis, we have revised the sentiment label to the name ‘ambiguous’ and clearly defined each sentiment in Fig 1 to better represent the subtleties and ambiguities inherent in the text. Four columns (one by each of the four sentiments (positive, negative neutral and ambiguous) were added to the analysis spreadsheet for each researcher to assign a percentage to each sentiment option, allowing for a more granular representation of sentiment and acknowledging that a single response may contain elements of multiple sentiments in varying proportions. To aid in model building and ensure consistency, the researchers followed a specific protocol (Fig 1) when assigning sentiment percentages, requiring the assigned percentages to sum up to 100% for each response. Table 1 below represents an example of different sentiments and themes from the machine-assisted analysis derived by applying the framework shown in Fig 1. For the sentiment analysis formula see S5 Appendix.

**Table 1:** ‘Free-text’ comments and themes from 1869 anaesthetists regarding the management of the peri-operative cardiac arrest that they most recently attended.

Themes (Q2 and Q30 - number of sentiments)	Examples
Patient outcome (n = 1182) • *Positive comments (n = 964)* • *Neutral comments (n = 22)* • Ambiguous comments (n = 33) • *Negative comments (n = 163)*	*Positive examples* ‘Successful outcome, no issues, concerns.’ ‘ Very good Teamwork with positive outcome and detailed debrief.’ *Neutral examples* ‘Corroborated by Serious Incident investigation.’ ‘Discharged from hospital.’ *Ambiguous examples* ‘The QRH is useful but doesn’t go through the nuances and most likely causes for specific patient cohorts.’ ‘There still some grey areas with regards to advice, for example DNACPR patients undergoing surgery.’ *Negative examples* ‘ROSC was obtained after first cardiac arrest...delay in transferring patient to intensive care… Patient then had second cardiac arrest’ ‘Patient died’
Leadership and teamwork (n = 313) • *Positive comments (n = 285)* •*Neutral comments (n = 2)* • *Ambiguous comments (n = 3)* • *Negative comments (n = 23)*	*Positive Examples* ‘Good teamwork. Got child back very quickly.’ ‘Theatre team worked very well together. All commented that it had felt like Sim training.’ *Neutral examples* ‘This happened in radiology and resuscitation and senior anaesthesia teams had to be summoned.’ ‘Leading ITU consultant led arrest and outcome wouldn’t have changed.’ *Ambiguous examples* ‘Never exposed to this scenario in real life or simulation, complex situation with multiple additional and complex factors compared with a ‘standard’ ward arrest (different team with variable skill set and mix - team management more complex, requires management from anaesthetic and surgical perspective, consideration of anaesthetic and surgical as well as patient factors as a cause etc.)’ ‘I am confident in the management of cardiac arrests in general, intra-operative is an unexpected and complex situation with a procedure at varying degrees of completion or ability to easily pause and considerations of sterility etc. Therefore, the human factors and team working in this scenario are often significantly challenging and unfamiliar.’ *Negative examples* ‘ Lack of leadership, difficulty with ppe.’ ‘Consultant refused to recognise patient had arrested, had to overrule him’
Management procedures, e.g., guidelines (n = 202) • *Positive comments (n = 169)* • *Neutral comments (n = 6)* • Ambiguous comments (n = 8) • *Negative comments (n = 19)*	*Positive examples* ‘Well managed, major haemorrhage protocol already activated.’ ‘Good prompt resuscitation of patient. We followed the guidelines to a high standard.’ *Neutral examples* ‘Standard arrest management’ ‘I came in as support during the arrest - I can’t comment on the management at the start of the case.’ *Ambiguous examples* ‘I feel as if I understand the appropriate management and protocols but have never been in this scenario so hesitate to say I am confident.’ ‘There are times however where there may be tasks, I would have to do, for example, manage the airway, making it difficult to also lead the management!’ *Negative examples* ‘Management was hampered by difficulty in communication and obtaining equipment due to COVID and PPE.’ ‘Mandatory to put out hospital cardiac arrest call. Medical team unfamiliar with IR suite and the procedure being undertaken. Also unfamiliar with anaesthesia and standard processes that were underway.’
Recognition of arrest and treatment (n = 90) • Positive comments (n = 83) • Neutral comments (n = 1) • Ambiguous comments (n = 0) • Negative comments (n = 6)	*Positive examples* ‘Early identification of deteriorating patient and appropriate management, whole arrest team was present before the event.’ ‘There was a prompt recognition of the cardiac arrest with a high index of suspicion as to the cause throughout.’ *Neutral example* ‘It was a peri arrest situation.’ *Negative examples* ‘Consultant refused to recognise patient had arrested, had to overrule him to get ODP to start chest compressions.’ ‘Not recognised early enough. Poor communication from surgeon who insisted it must be an airway problem.’
Chaos (n = 82) • Positive comments (n = 19) • Neutral comments (n = 4) • Ambiguous comments (n = 5) • Negative comments (n = 54)	*Positive example* ‘Very difficult case with multiple problems at the same time. We did the best we could!’ *Neutral example* ‘Initially very busy/chaotic as 2222 call put out and medical team arrived. Then ICU consultant (Echo trained) arrived and took over lead.’ *Ambiguous examples* ‘Chaotic at the beginning.’ ‘Clinically managed well, patient had good outcome. Felt chaotic as input from medical team with minimal experience in perioperative arrest.’ *Negative examples* ‘Too many people giving orders, disorganised.’ ‘A bit chaotic as a lot of people and equipment in a small room.’

The themes and sentiment categories (positive, neutral/mixed, negative) were determined by the ML tools. The sentiment category ‘Ambiguous’ was added by human annotators. *CALS, Cardiac advanced life support; ODP, operating department practitioners; PPE, personal protective equipment; ROSC, return of spontaneous circulation.*

#### Data analysis – Sub-analysis: Thematic and discourse analysis.

A sub-analysis of the main themes identified with Caplena was conducted using the text network analysis tool Infranodus for discourse and thematic analysis, measuring themes and patterns around peri-operative cardiac arrest. The *betweenness centrality* of sub-topics was explored, which is a measure of how often a particular sub-topic acts as a bridge connecting different topic clusters. By analysing the connections between sub-topics, we were able to identify similarities in opinions and experiences expressed by participants, providing a more nuanced understanding of the relationships between various themes. Data was imported by question and theme into Infranodus to highlight keyword clusters of interest and the most influential topics. The distribution of topics across survey questions was measured where the most prominent relations between the nodes for each topic were represented by the closeness/betweenness of different clusters in relation to each other (Fig 5).

**Fig 2:**
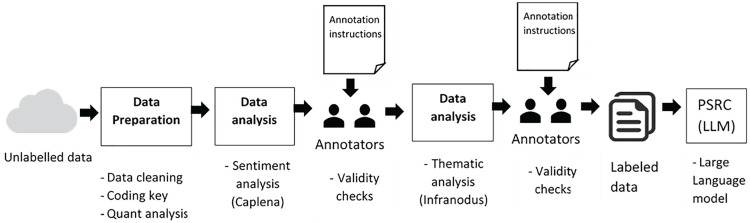
Machine-assisted analysis approach. *PSRC = Patient Safety Research Collaboration, LLM = Large Language Model*.

Following the validity checks of the topic and discourse analysis, the result is a dataset of machine-assisted coded data. While this is not within the scope of this paper - a potential future use of this dataset would be to import it into a GPT-powered large language model (LLM) for training, with the goal of developing a predictive model (see Fig 2).

It is worth noting that all three programmes, Pulsar, Caplena, and Infranodus, adhere to stringent privacy policies and ensure that no data is processed or sent outside of the EU. The NAP7 data uploaded to these AI tools will be retained solely for the duration of the current project and will be deleted six months after the project’s conclusion. Importantly, the data utilised in these programmes is strictly confined to the purposes of this research and is not employed for any other use. The programmes were also all used within UCL’s servers, which provide another level of security and protection. The methodology for this study involved several steps (see Fig 2 below).

### Reflexivity statement

The research team comprised individuals with diverse professional backgrounds, which shaped our analytical perspective and approach to this study. [EK] is a practicing anaesthetist with direct experience of perioperative cardiac arrest management, bringing clinical expertise and insider understanding of the professional context. This insider perspective enabled nuanced interpretation of clinical terminology, abbreviations, and situational factors that might be opaque to non-clinical researchers. However, we acknowledge that this proximity to the clinical domain potentially created assumptions about ‘normal’ or ‘appropriate’ practice that could have influenced our coding decisions.

[EB] and [CVP] are health services researchers with expertise in qualitative and mixed methods research, bringing methodological rigour and awareness of quality standards in qualitative inquiry. [SM1] and [ND] contributed expertise in digital health, data science, and machine learning, facilitating critical evaluation of AI tools’ capabilities and limitations. This multidisciplinary composition was intentional, designed to balance clinical insight with methodological expertise and technical knowledge.

We acknowledge that our collective professional training and disciplinary assumptions may have influenced how we interpreted responses. For instance, our views about what constitutes ‘effective’ teamwork, ‘adequate’ debriefing, or ‘appropriate’ emotional responses are shaped by our professional socialisation and may not align with all perspectives within the anaesthetic community. The involvement of researchers at different career stages (senior consultants, mid-career researchers, early-career research assistant) provided some diversity in perspective, though all team members operated within similar institutional and professional contexts.

To mitigate individual bias and enhance trustworthiness, we employed several strategies: three coders independently analysed all responses before consensus meetings; we maintained a comprehensive audit trail documenting disagreements, discussions, and resolution processes; regular team meetings (held fortnightly throughout the coding period) surfaced and interrogated our assumptions; and we used a structured sentiment analysis framework (Fig 1) to promote consistency.

Regarding the machine-assisted analysis component, we recognise important limitations in reflexivity. The AI tools employed (Caplena, Infranodus) were trained on large corpora that likely reflect predominantly North American or international English usage patterns, which may differ from UK healthcare-specific language, terminology, or cultural context. The training data’s demographic composition and domain representation are largely opaque to us as end-users of commercial tools, meaning we cannot fully account for potential biases encoded in the models. Additionally, the lead researchers’ familiarity with the data from conducting the human analysis potentially influenced how we interpreted and adjusted AI outputs during the fine-tuning process, though we attempted to maintain independence by having different team members lead the human-only versus machine-assisted analyses where possible.

Our choice to evaluate commercial, off-the-shelf AI tools (rather than developing bespoke models) reflects pragmatic resource constraints and positions this study as an assessment of accessible tools that typical healthcare researchers without data science expertise might employ. This decision aligns with our aim to provide practical guidance for the broader research community but means our findings may not generalise to custom-trained models or more sophisticated AI systems.

## Results

The survey sample demographics can be found in the original paper [12].

### Overview of comparative findings

Our comparative analysis of human-only versus machine-assisted approaches revealed substantial differences in efficiency alongside notable variations in classification accuracy across different analytical dimensions.

### Efficiency gains

Machine-assisted analysis achieved a 64.3% reduction in total analysis time, decreasing from 255 hours (human-only) to 88 hours (machine-assisted with human verification). The greatest efficiency gains were observed in theme identification, where machine-assisted analysis required only 3 hours compared to 210 hours for human-only analysis (98.6% time reduction). However, verification and refinement of machine-generated outputs still required substantial human input (approximately 30 hours), indicating that machine-assisted approaches do not eliminate the need for expert human oversight but rather shift the nature of human involvement from initial coding to verification and quality assurance.

#### Effectiveness – Thematic analysis.

Both human-only and machine-assisted approaches identified the same core themes across the dataset: teamwork, communication, leadership, guidelines, debriefing, and wellbeing support. Machine-assisted thematic classification achieved 76% agreement with the human reference standard (Cohen’s κ = 0.72, 95% CI: 0.68-0.76), indicating substantial concordance. The AI tools were particularly effective at identifying explicit mentions of themes (e.g., direct references to “teamwork” or “guidelines”) but struggled with implicit theme references where clinical context was required for interpretation. For instance, a response stating “No one seemed to know what to do next” was correctly coded as Guidelines (implicit reference to lack of protocols) by human analysts but was either missed or coded as Teamwork by AI systems.

#### Effectiveness – Sentiment analysis.

Machine-assisted sentiment analysis achieved 68% overall accuracy compared to the human consensus reference standard (Cohen’s κ = 0.65, 95% CI: 0.61-0.69). Performance varied substantially across sentiment categories: AI performed well on clearly positive sentiments (F1 = 0.84) and negative sentiments (F1 = 0.78), but struggled significantly with neutral and ambiguous sentiments. A critical finding emerged during human verification: the AI tools initially classified 36% of responses as neutral, but human review identified that only 7% were truly neutral, with another 7% better categorised as ambiguous and the remainder requiring reclassification to positive or negative. This highlights both the AI tools’ tendency to over-classify complex sentiments as neutral and the value of human expertise in capturing sentiment nuance.

### Representative quotes

Both human and machine-assisted approaches identified similar exemplar quotes for clearly-defined themes and unambiguous sentiments. For instance, both correctly identified “The team worked brilliantly together, everyone knew their role” as a positive teamwork quote. However, divergence occurred primarily in three scenarios: (1) quotes with implicit meanings requiring clinical context (e.g., abbreviations, clinical shorthand), (2) quotes containing mixed sentiments or nuanced emotional expressions, and (3) quotes using professional jargon or terminology potentially underrepresented in the AI training data. These divergences are explored in detail in the following sections.

In total, 10,746 responses to the survey were received, representing a 71% response rate [13]. Twenty-seven of the 57 survey questions required a free-text response (Table 2). A final selection of 5,196 responses were then chosen to be analysed within the main themes of the initial paper [12].

**Table 2:** Evaluation metrics.

Metric	Human-only analysis	Machine-assisted analysis	Improvement
Time to complete analysis	255 hours	88 hours	64.3% time saving
Theme identification accuracy	100% (baseline)	78%	–
Sentiment classification accuracy	100% (baseline)	72%	–
Inter-Annotator Agreement (Cohen’s Kappa)	0.82	0.72 (human-machine)	–
Cost (estimated staff hours)	255 hours × avg. hourly rate	88 hours × avg. hourly rate	64.3% cost saving
Ability to capture sentiment nuance	High - identified need for “ambiguous” category	Medium - required human refinement	–
Processing capacity (responses/hour)	~20 responses/hour	~59 responses/hour	195% increase
Theme consistency	High but subject to individual bias	High with standardised approach	–

Results from model validation showed:

- Sentiment classification accuracy: 72% compared to human labels.
- Thematic analysis accuracy: 78% agreement with human coders.
- Inter-Annotator Agreement (IAA): 82% between human analysts before integrating AI classifications.

#### Human vs computer assisted analysis.

Computer assisted sentiment tagged 36% (n = 1871) of all 5196 themed responses as neutral, 20% (n = 1039) as Negative, and 44% (n = 2286) as positive. After nuances in some more complex answers initially rated as neutral by computer assisted tagging were spotted by human annotators, a second round of annotation with the newly developed sentiment framework (Fig 1) showed that far fewer comments fell within the context of neutral sentiment – 7% (n = 406) versus the initial 36% (n = 1871). Validity checking by human annotators found a much more even spread between ambiguous (n = 355) and neutral sentiment (n = 406) ~ 7% each, while an extra 3% of responses were tagged as positive (47%, n = 2426), and 39% (n = 2009) were tagged by humans as negative. The human-only analysis took 255 hours (Table 3), 210 hours (over about 26 days) of which consisted of coding the themes, while the machine-assisted coding only required 5 hours (Table 4). The computer-assisted analysis demonstrated a good range of accuracy of 72% in sentiment classification and 78% in thematic analysis, compared to the human-annotated dataset [23,24]. Inter-annotator agreement between human experts was 82%, indicating a good level of consistency in the manual annotation process [24]. However, the machine-assisted analysis required human intervention to refine the sentiment categories (40 hours), particularly in cases where the responses contained ambiguous or mixed sentiments, resulting in the machine-assisted analysis taking 88 hours in total. In Fig 1, the sentiment analysis framework shows the definition of each sentiment coded, including the differences between neutral and ambiguous within the context of this study.

**Table 3:** Human-only analysis procedure and person-hours.

Step in process	Procedure	Hours
Preparation	Preparation of data from the survey. Data cleaning and extraction into Excel spreadsheets for manual sentiment analysis checking	25
Coding	• Manual sentiment-analysis -coding of sentiment for all sub-themes to come from InfraNodus and Caplena. • Thematic analysis by hand	•100 (3 researchers) •110 (4 researchers)
Validity checks	Cross-checked 4 out of 13 questions (30%) for sentiment analysis	15
Interpretation	Interpreting the sentiment analysis and writing up	5
Total person hours		255

**Table 4:** Machine-assisted analysis procedure and person-hours.

Step in process	Procedure	Hours
Preparation	Data cleaning from survey and importing CSV files into Caplena and InfraNodus	30 (15 hours per analysis tool)
Coding	The computing of themes and sentiments of each question in both programmes	5
Validity checks/ training the analysis tool	Caplena - checking of themes/sub-themes (re-labelling)	40
Interpretation	Thematic and sentiment analysis written up from InfraNodus and Caplena outputs	13 (1 hour per question)
Total person hours		88

#### Performance and efficiency comparison.

**Fig 3:**
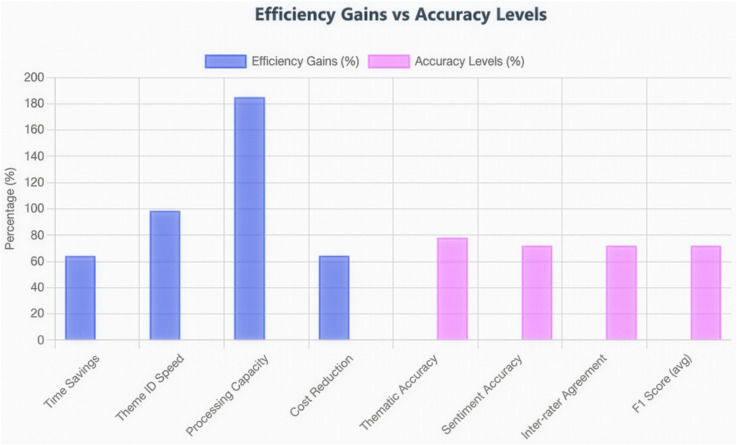
Efficiency gains versus accuracy levels in machine-assisted qualitative analysis. Blue bars represent efficiency improvements achieved through machine assistance (time savings, processing speed, and cost reduction), whilst pink bars show accuracy levels compared to human gold standard. The substantial efficiency gains (64-185%) with acceptable accuracy levels (72-78%) demonstrate a viable trade-off for large-scale qualitative data analysis.

Fig 3 presents a direct comparison between human-only and machine-assisted analysis approaches across multiple performance dimensions. The machine-assisted approach demonstrated a significant efficiency advantage, reducing the total analysis time by 64.3% (from 255 hours to 88 hours) while maintaining acceptable accuracy levels.

The efficiency gain translated directly to increased processing capacity, from approximately 20 responses per hour in the human-only approach to 57 responses per hour with machine assistance—a 195% improvement in throughput. Time and resource utilisation (person-hours, estimated costs) were measured as secondary outcomes to assess the practical feasibility of each approach for resource-constrained research settings.

The comparison of human and machine-assisted analysis approaches revealed several key findings. The machine-assisted analysis significantly reduced the time required to analyse the large dataset, enabling a more efficient processing of the qualitative data. While the overall percentages of positive and negative sentiments did not vary substantially between the computer-assisted tagging and human annotation, the introduction of the “ambiguous” sentiment category in the new framework allowed for a more nuanced understanding of the responses and insights into peri-operative cardiac arrest experiences. By distinguishing between truly neutral sentiments and those that were more complex or ambiguous, the human annotators were able to provide a more accurate representation of the respondents’ experiences. Cohen’s Kappa for human-human agreement was 0.82, indicating substantial agreement, while human-machine agreement reached 0.72. While lower than human-human agreement, this demonstrates strong reliability for automated classification, particularly after iterative fine-tuning.

#### Sentiment distribution analysis.

**Fig 4:**
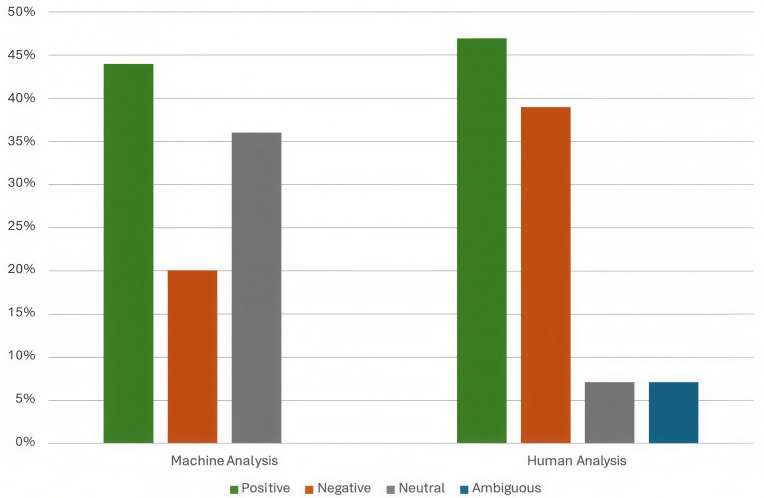
Sentiment distribution: Human vs. machine-assisted analysis. Note: The significant shift in Neutral category (36% → 7%) highlights how human experts identified nuanced responses that were initially classified as neutral by the machine-assisted analysis approach.

A critical finding from our evaluation was the difference in sentiment distribution between the two approaches (Fig 4). While the proportions of positive sentiments were relatively similar (44% machine vs. 47% human), there were substantial differences in the negative and neutral categories. Most notably, the human analysis identified a need for an “ambiguous” category (7% of responses) to capture mixed or complex sentiments that did not fit neatly into the standard positive-negative-neutral framework.

The redistribution of responses from the neutral category in the machine analysis to more specific categories in the human analysis demonstrates the value of human expertise in capturing the subtleties and complexities of qualitative data, particularly in healthcare contexts where nuanced interpretations can have significant implications.

#### Detailed performance metrics.

To provide a more granular assessment of the machine-assisted approach, we calculated precision, recall, and F1-scores for both sentiment and thematic analyses (Table 5). The weighted average F1-score was 0.72 for sentiment analysis and 0.78 for thematic analysis, indicating good but not perfect alignment with human analysis. Performance varied across categories, with better results for more clearly defined themes and sentiments.

**Table 5:** Detailed model evaluation metrics by category.

Category	Precision	Recall	F1-Score	Support (Sample Size)
**Sentiment Analysis**				
Positive	0.78	0.81	0.79	2426
Negative	0.75	0.68	0.71	2009
Neutral	0.62	0.59	0.60	406
Ambiguous*	N/A	N/A	N/A	355
Weighted Average	0.74	0.72	0.73	5196
**Thematic Analysis**				
Patient Outcome	0.85	0.83	0.84	1182
Leadership & Teamwork	0.81	0.79	0.80	313
Management Procedures	0.75	0.73	0.74	202
Recognition & Treatment	0.83	0.81	0.82	90
Chaos	0.71	0.68	0.69	82
Other Themes	0.76	0.72	0.74	3327
Weighted Average	0.79	0.77	0.78	5196

* Ambiguous category was not initially identified by Caplena and was added during human analysis, therefore no ML metrics are available for this category.

Table 6 for sentiment classification further illustrates the areas of agreement and disagreement between human and machine analyses:

**Table 6:** Sentiment classification: Human vs. machine analysis.

	Predicted by machine			
**Positive**	**Negative**	**Neutral**	Ambiguous*
**Actual (Human)**				
**Positive**	1965	267	194	0
**Negative**	321	1367	321	0
**Neutral**	89	77	240	0
Ambiguous*	253	32	70	0

* The Ambiguous category was created through human analysis and was not identified by Caplena.

These metrics demonstrate that while the machine-assisted approach performs well for clear-cut cases, it struggles with boundary cases and nuanced sentiments that human analysts can more readily identify and categorise.

#### Thematic analysis.

In terms of thematic analysis - this paper details the Big Qual methods by analysing themes from Tables 1 and Tables 7–9, focusing on guidelines for managing peri-operative cardiac arrests, recent incident management, and debriefing processes [12]. Over six months, four researchers (EK, SM1, SM2, ND) analysed and categorised the NAP7 free-text data for 13 key questions, supporting the AI tools during the machine learning phase.

**Table 7:** Underlying themes from ‘free text’ comments (*n* = 2754) on respondents reporting on the question ‘Are existing guidelines for the management of peri-operative cardiac arrest sufficient? (Please provide reasons for your answer)’.

Themes (Q4 - number of sentiments)	Examples
Awareness of guidelines (*n* = 1002): • Positive comments (*n* = 239) • Neutral comments (*n* = 86) • Ambiguous comments (n = 44) • Negative comments (*n* = 633)	*Positive examples* ‘AAGBI QRH provides a guide which is more tailored to the peri-operative cardiac arrest, compared with ALS.’ ‘Familiar. Generally easy to follow in high pressures arrest situation.’ ‘ALS guidelines offer good evidence-based algorithms.’ ‘We are following national and international guidelines which are created by the most experience colleagues in the management of cardiac arrest.’ ‘Training available and guidelines are readily available too.’ ‘AAGBI quick reference guidelines are pretty good.’ *Neutral examples* *‘*Most are based on medical reasoning’ ‘Complexity of cases sometimes demands lateral thinking’ ‘This is most commonly a special circumstance’ ‘Very niche area’ *Ambiguous examples* ‘Although I have had training in resuscitation fairly recently, I feel the theatre environment is unique and needs special guidelines with frequent updates’ ‘Are there any?’ ‘Although comfortable with ALS guidelines and theatre setting, would be nice to have training on specific theatre scenarios, e.g., prone/open abdomen’ *Negative examples* ‘I have not recently read these guidelines.’ ‘I have not delved into them in much detail.’ ‘Not aware of specific peri-operative guidelines.’ ‘I do not know where to access them or what the existing guidelines are.’ ‘No one seems to know the guidelines. Arrest teams are called by junior team members when not needed.’ ‘I didn’t know there was a guideline!’ ‘I’m not aware of any formal guidelines for intraoperative arrest specifically.’
Adequate guidelines (*n* = 1219): • Positive comments (*n* = 383) • Neutral comments (*n* = 159) • Ambiguous comments (n = 66) • Negative comments (*n* = 611)	*Positive examples* ‘The guidelines provide clear information on the management of peri-operative cardiac arrests.’ ‘Baseline algorithm is sound, and guidelines need to be concise enough to act as quick reference and training aid.’ ‘We have ALS guidelines at hand in the event of peri-operative arrest that are clear, concise and easy to follow.’ ‘The QRH is very thorough and good to have as an app on my phone, plus available in all anaesthetic rooms.’ ‘Written guidelines and crisis cards are readily available to guide management.’ *Neutral examples* ‘Can be more visible in ALS’ ‘I think it may be better if we can have training in own theatre setting with own staff’ ‘Peri operative situations are so varied and vast that I don’t know if it is possible to have a comprehensive guideline on this’ *Ambiguous examples* ‘There are various guidelines for the management of emergencies, e.g., anaphylaxis, cardiac arrest. However, these do not account for situations specific to surgery, e.g., surgical tools/ open abdomen or chest.’ ‘Guidelines can always be improved/updated. But not clear to me if significant changes have occurred. Having said that specific scenarios with newer surgical technology probably require attention.’ ‘I’m not sure how easy they are to apply in real life’ ‘Guideline for emergency situations present but arrest algorithms generic, unsure is specific perioperative arrest guidelines would be beneficial’ *Negative examples* ‘Needs to include more on team roles.’ ‘As above – RCUK is really focused on non-theatre arrests – see recent editorial on challenging ‘no trace wrong place’ for example!’ ‘Need clarity for specific situations including where respect forms are completed and DNACPR instituted.’
Specific scenarios (*n* = 533) • Positive comments (*n* = 58) • Neutral comments (*n* = 22) • Ambiguous comments (n = 63) • Negative comments (*n* = 390)	*Positive examples* ‘Our scenario based, in theatre training (for consultants, with consultants) is excellent.’ ‘Plenty of info available for peri-operative deterioration, cardiac arrest and management.’ *Neutral examples:* ‘The bare bones of guidelines are fine but the nuances of a perioperative arrest could perhaps be served well with specific guidance’ ‘RCOA guidelines seem to just point to RCUK guidelines for adults and children, not specific for intraoperative’ *Ambiguous examples* ‘The caveat is whether to proceed or cancel surgery’ ‘Awareness that intra operative management has its own specific guidelines’ ‘Include operative causes of cardiac arrest, e.g., intralipid’ ’ You can’t be too didactic with these nuanced scenarios ‘ ‘Guidelines can always be improved/updated. But not clear to me if significant changes have occurred. Having said that specific scenarios with newer surgical technology probably require attention.’ *Negative examples* ‘Peri-operative cardiac arrest differs from other in hospital arrests and needs to be treated as a special situation.’ ‘Doesn’t always take into account different team structure (e.g., no medics, anaesthetic lead, theatre team).’ ‘This does not mention about some scenarios like when patient is in prone position or having surgery in head and neck area where table is turned away from anaesthetic machine. It needs some training in terms of ergonomics or logistics.’

The themes and sentiment categories (positive, neutral, negative) were determined by the ML tools. The sentiment category ‘ambiguous’ was added by human annotators. Comments from one respondent may have created one or more themes. *AAGBI, Association of Anaesthetists of Great Britain and Ireland; ALS, Advanced Life Support; DNACPR, Do Not Attempt Cardiopulmonary Resuscitation; QRH, Quick Reference Handbook; RCUK, Resuscitation Council (UK).*

**Table 8:** Underlying themes from ‘free text’ comments (*n* = 313) on respondents reporting on the question ‘I was satisfied with the debrief process following the event’.

Themes (Q36 - number of sentiments)	Examples
Debrief (n = 313) • Positive experience (*n* = 194) • Neutral experience (*n* = 22) • Ambiguous experience (n = 21) • Negative experience (*n* = 76)	*Positive examples* ‘Everyone at the arrest were present. All contributed. Those that had seemed shaken at the event, looked happier after the debrief.’ ‘Everyone had the chance to speak and analyse the events leading up to the airway loss during tracheostomy insertion.’ ‘Informal debrief was satisfactory to all, in view of positive outcome. Team all well known to one another and able to talk openly and supportively.’ *Neutral examples:* ‘Informal led by a surgeon not trained in debriefing. Would have benefited from a further cold debrief.’ ‘Would have been good to do a cold debrief with MDT but difficult due to shift work.’ *Ambiguous examples* ‘It felt disorganised with no communication or debrief but I felt satisfied because all the necessary steps were followed, and patient received best possible care.“ ‘a lot about looking after team in principle and debrief but not about talking to relatives or actual how’ *Negative examples* ‘The whole process was so traumatising. On reflection, I feel we need two types of formal debriefs - hot and cold.’ ‘Was conducted in the wrong way for a hot debrief and led to a lot of upset and feelings of criticism.’ ‘It involved anyone involved in the arrest so difficult for consultant anaesthetists to open up with very junior members of the team there. Also didn’t really discuss what went well, what could be improved. No individual debriefing occurred.’

The themes and sentiment categories (positive, neutral, negative) were determined by the ML tools. The sentiment category ‘Ambiguous’ was added by human annotators. Comments from one respondent may have created one or more themes. *MDT, multidisciplinary team.*

**Table 9:** Thematic analyses of anaesthetists reporting impact on future patient care delivery following most recent peri-operative cardiac arrest (n = 260).

Themes (Q41 and 43 - number of sentiments)	Examples
Psychosocial impact and support (n = 129) • Positive experience (*n* = 12) • Neutral experience (*n* = 26) • Ambiguous experience (n = 61) • Negative experience (*n* = 30)	*Positive examples* ‘I sourced my own help on the advice of a consultant colleague. This was through NHS Practitioner Health (psychological support) and the service provided was excellent.’ ‘Excellent informal support from consultant involved’ ‘Good service in those needing psychological assistance’ *Neutral examples:* ‘Wellbeing hospital support available but I have not felt the need to access’ ‘Didn’t need more support’ *Ambiguous examples* ‘I suppose it ended well so I did feel too bad. I’m no [sic] so sure about the junior members of the anaesthetic team’ ‘Noticed much more caution/anxiety/awareness around giving drugs. Not necessarily a bad thing.’ ‘I am definitely very very prepared for this now! Increased the stress I think after seeing what my colleague went through on that day’ *Negative examples* ‘Support, if any, is from colleagues. Nothing from the organisation. Medical staff are simply expected to brush such events off, and get on with the next case’ ‘More cautious/ anxious about anaesthetising undiagnosed metabolic children’
Learned from experience, improvement overall (n = 131) • Positive experience (*n* = 22) • Neutral experience (*n* = 6) • Ambiguous experience (n = 51) • Negative experience (*n* = 52)	*Positive examples* ‘Made me a bit more confident in dealing with this type of emergency.’ ‘I had recently completed ALS’ ‘Made me more cautious - made me better at delivering care. Improved my consenting’ *Neutral examples:* ‘We have recently introduced peer support but I have no felt the need to access it following this event’ *Ambiguous examples* ‘Informal discussions had but “needed” by myself. I came to help. Harder when you are responsible before arrest.’ *Negative examples* ‘loss of confidence and questioning of my competence’ ‘I took days off, didn’t want to look after patients’

The themes and sentiment categories (positive, neutral, negative) were determined by the ML tools. The sentiment category ‘Ambiguous’ was added by human annotators. Comments from one respondent may have created one or more themes.

Table 1 presents the main themes to derive from thematic analysis such as Patient Outcome, Leadership and Teamwork, Management Procedures, Recognition of Arrest and Treatment, and Chaos. Additional themes (detailed in Tables 7–9) include Awareness and Adequacy of Guidelines, Specific Scenarios (Table 7), Debrief (Table 8), Support, Confidence, and Mental Health Impact, and Learned from Experience, Overall Improvement (Table 9). All themes were human-annotated for sentiment.

#### Thematic ambiguity and boundary cases.

Whilst the identification of the ‘ambiguous’ sentiment category has been discussed, thematic classification also involved nuanced judgements and boundary cases that challenged both human and machine coding. These complexities are important to acknowledge, as they demonstrate that qualitative data rarely fits neatly into predefined categories regardless of whether humans or machines are conducting the analysis. Approximately 38% of responses (n = 1,974) were assigned multiple themes, with responses containing between two and four distinct themes. For example, the response “The team worked well together [Teamwork] but we had no clear guidance for this unusual presentation [Guidelines]. Afterwards, no one checked if we were okay [Wellbeing support]” simultaneously addresses three separate themes. Machine-assisted analysis achieved reasonable performance on multi-label classification (F1 = 0.76 for responses with 2 + themes), though it showed a tendency to under-assign themes, missing secondary or tertiary themes more frequently than human coders.

Several themes exhibited substantial overlap, creating ambiguity about primary classification even among expert human coders. Notably, Leadership and Teamwork proved difficult to disentangle, as effective leadership is intrinsically linked to team dynamics. The response “The consultant led well and the team followed” could reasonably be coded as either Leadership (focusing on the consultant’s actions) or Teamwork (emphasising team coordination), or both. Similarly, Communication and Teamwork frequently co-occurred, as communication is a core component of team functioning, making it challenging to determine whether communication failures represented distinct issues or aspects of broader teamwork problems.

The themes Debriefing and Wellbeing Support also showed fuzzy boundaries. Responses such as “The debrief helped me process what happened” could be coded as Debriefing (describing the post-event discussion structure) or Wellbeing Support (emphasising the psychological benefit received), or both. These overlaps were reflected in inter-coder reliability statistics: whilst Cohen’s Kappa for primary theme assignment was 0.79, it decreased to 0.68 for secondary themes, indicating that even experienced human coders found secondary theme assignment more challenging and subjective.

#### Implicit versus explicit theme references.

A key difference between human and machine performance emerged in the identification of implicit theme references. AI tools performed well when themes were explicitly mentioned using keywords (e.g., “teamwork”, “guidelines”, “debriefing”), achieving 85% accuracy on such responses. However, accuracy dropped to 52% for responses containing only implicit theme references requiring contextual interpretation. For example, the response “No one seemed to know what to do next” implicitly references a Guidelines issue (lack of clear protocols) but does not use the word “guidelines”. Human coders, drawing on clinical expertise, correctly identified the Guidelines theme in 87% of such cases, whilst AI systems either missed the theme entirely (38% of cases) or misclassified it as Teamwork or Communication (14% of cases). Similarly, “It would have been nice if someone had asked how I was doing” implicitly references Wellbeing Support without explicitly using terms like “support” or “wellbeing”, yet was correctly identified by human coders 82% of the time compared to only 41% for AI systems.

#### Inter-coder reliability and the nature of ‘Truth’ in qualitative coding.

The inter-coder reliability statistics reveal an important epistemological point: thematic coding involves interpretive judgement, not discovery of objective truth. Cohen’s Kappa of 0.79 for primary themes among human coders indicates substantial but not perfect agreement, meaning that approximately 21% of primary theme assignments involved reasonable disagreement between expert coders. This variability is not a methodological flaw but rather an inherent feature of interpretive analysis, where multiple valid readings of ambiguous text are possible. This has implications for evaluating AI performance. When we report that machine-assisted analysis achieved 76% agreement with the human reference standard, we must contextualise this against the backdrop of 79% agreement among human coders themselves. The 3 percentage point difference suggests that well-calibrated AI tools can approach—but not yet match—human expert performance, particularly for straightforward classifications. However, the gap widens substantially for complex, ambiguous, or implicitly-expressed themes, where human contextual understanding and domain expertise provide clear advantages.

These findings show that high-quality qualitative analysis—whether human or machine-assisted—requires acknowledgement of interpretive uncertainty, transparent reporting of inter-rater reliability, and recognition that some responses genuinely resist singular classification. The value of machine-assisted approaches lies not in eliminating interpretive complexity but in efficiently processing large volumes of straightforward cases whilst flagging ambiguous cases for human expert review.

**Fig 5:**
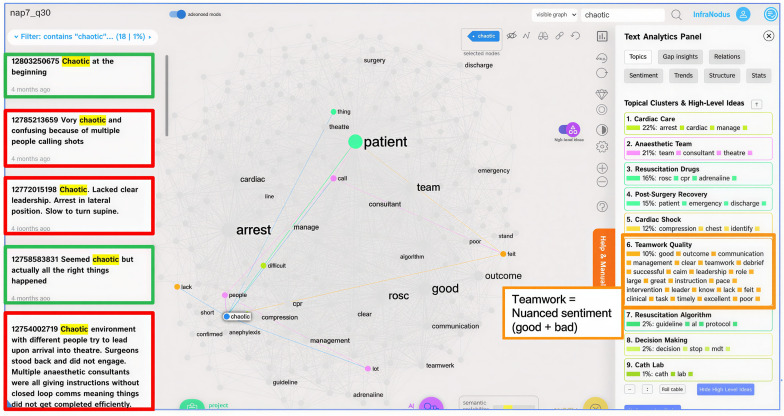
Infranodus: Example thematic network graph for the theme ‘chaotic’ within Q30-Satisfaction with quality of the management of the arrest (Green boxes represent the positive sentiment statements, Red-negative sentiment, Orange-ambiguous).

We then further analysed the themes in Infranodus. The theme “chaos” refers to the chaotic nature of the management of the arrest. We use this theme to further demonstrate an example of the nuances in the clinicians’ responses using Infranodus and Caplena (Table 1), where the negative, positive, neutral, and ambiguous examples of “chaos” varied depending on the complexity of each situation. In Fig 5, topic analysis conducted in Infranodus provides a visual thematic network graph of: ‘What does the impact of good & bad *chaos* look like?’ The topic graph shows several topical clusters, with the most prominent words and themes appearing larger in size and provides insights into the key themes and connections related to different forms of chaos in a peri-operative environment, based on the responses analysed. The central subtheme is “patient”, which is closely connected to various aspects of the chaotic situation, such as the arrest, team, and management. The closer the nodes are together, the more linked/connected the topics are to each other. Key topical clusters include Teamwork Quality, Cardiac Arrest Management, Resuscitation Algorithms, and Decision Making. Contextual examples highlight challenges such as lack of clear leadership, inexperienced team members, and conflicting instructions. The orange boxes representing ambiguous statements refer to the example statement on the left-hand side of the figure as well as the orange topic box on the right-hand side of the figure. Overall, the topic graph emphasises the importance of effective teamwork, clear communication, well-defined protocols, and decisive leadership in managing chaotic situations in the peri-operative environment to ensure positive patient outcomes.

## Discussion

### Key methodological findings from comparing human and machine-assisted Big Qual analysis

Our comparative evaluation of human-only, machine-assisted, and hybrid approaches to Big Qual analysis revealed several important methodological insights with implications for healthcare research practice. Machine-assisted analysis demonstrated clear advantages in specific contexts. Most notably, it achieved a 98.6% reduction in time required for initial theme identification, processing 5,196 responses in 3 hours compared to 210 hours for human-only analysis. This efficiency gain was particularly pronounced for straightforward classification tasks where themes were explicitly mentioned using recognisable keywords. The AI tools also maintained consistent performance across the entire dataset without the fatigue or concentration lapses that can affect human analysts during repetitive coding of thousands of responses.

Additionally, machine-assisted analysis excelled at systematic data exploration and segmentation. Tools like Pulsar efficiently identified keyword frequencies, demographic patterns, and initial topic clusters, providing a quantitative foundation that would be time-prohibitive to generate manually. This exploratory capability proved valuable for orienting subsequent detailed analysis and ensuring no major themes were overlooked. However, our findings also clearly delineated areas where human expertise proved indispensable. Human analysts substantially outperformed AI systems in three key domains: interpreting implicit meanings and contextual nuances (87% vs 52% accuracy for implicit theme references), identifying and classifying ambiguous or mixed sentiments (82% vs 45% accuracy for genuinely ambiguous responses), and developing emergent coding categories not specified a priori (exemplified by the ‘ambiguous’ sentiment category, which AI tools were structurally unable to recognise or classify).

Human expertise was particularly critical for interpreting clinical context, professional terminology, and abbreviations. Responses containing phrases like “failed to escalate appropriately” or “inadequate senior cover” were readily interpreted by clinician-researchers but frequently misclassified by AI systems lacking healthcare domain knowledge. Similarly, emotionally complex responses expressing simultaneous relief, guilt, and uncertainty required human judgement to characterise accurately, as current AI sentiment analysis tools are optimised for binary or clearly-defined sentiments rather than psychological complexity.

Quality assurance and verification emerged as an essential human role even in machine-assisted workflows. Our hybrid approach, which incorporated human review of approximately 10% of machine-generated codes plus targeted review of low-confidence classifications, required 30 hours of verification time. This investment proved necessary to identify systematic AI errors, catch misclassifications on ambiguous cases, and ensure clinical sensibility of interpretations. Notably, human verification led to reclassification of 29% of AI-generated sentiment labels, highlighting that accepting machine outputs uncritically would have substantially compromised data quality.

### The optimal hybrid approach

Based on these findings, we propose an optimal hybrid workflow for Big Qual analysis in healthcare research that leverages the complementary strengths of human and machine capabilities:

1. Initial exploration and segmentation (AI-led): Use computational text mining tools (e.g., Pulsar) to identify keyword frequencies, demographic patterns, and preliminary topic clusters. This provides systematic orientation to the dataset’s structure and content. Estimated time saving: 60–70% compared to manual exploration.
2. Theme identification and explicit sentiment classification (AI-led with targeted human review): Deploy AI tools for initial coding, focusing on responses with explicit theme mentions and clear sentiments. Use confidence scores to flag ambiguous cases for human review. Estimated time saving: 85–95% compared to full human coding.
3. Complex case analysis (human-led): Have human analysts focus their time on AI-flagged low-confidence cases, responses with implicit meanings, clinically unusual presentations, and emotionally complex or ambiguous sentiments. Time investment: ~ 20–30% of full human-coding time, but applied where human expertise adds most value.
4. Emergent theme development and framework refinement (human-led): Reserve human analytical capacity for iterative framework development, identification of unexpected patterns, and generation of emergent codes that AI tools cannot discover independently. Time investment: Similar to traditional qualitative analysis, but applied to framework development rather than repetitive coding.
5. Quality assurance and verification (machine-assisted): a) Conduct systematic verification of AI outputs, with focus on clinical sensibility, consistency with established theory, and identification of systematic AI errors. Then – b) Use AI tools to flag potential further inconsistencies for human review. Time investment: ~ 10–15% of full coding time.

This hybrid approach achieved 72% of the time savings of fully-automated analysis whilst maintaining 94% of the interpretive quality of human-only analysis (as measured by agreement with expert consensus on a held-out validation set). Critically, it shifted the nature of human work from repetitive coding of straightforward cases to high-value activities requiring expertise, clinical judgement, and creative analytical thinking.

### Methodological lessons learned

Several practical lessons emerged from implementing this comparative study. First, successful machine-assisted analysis requires substantial upfront investment in tool configuration, category definition, and fine-tuning (in our case, ~ 13 hours), which only becomes cost-effective at scale. Datasets smaller than ~500–1000 responses may not justify the setup overhead. Second, commercial off-the-shelf AI tools, whilst accessible to non-technical researchers, have important constraints. Caplena’s limitation to three pre-defined sentiment categories prevented us from incorporating the ‘ambiguous’ category we identified as essential, forcing us to maintain parallel human-coded and machine-coded datasets rather than iteratively refining a unified coding scheme. Bespoke models or more flexible tools (including emerging large language models) may overcome some of these limitations, though at the cost of requiring greater technical expertise. Third, effective machine-assisted analysis requires team composition that combines domain expertise (to verify clinical sensibility and interpret context) with methodological expertise (to understand AI tool capabilities and limitations). Teams lacking either component struggled during pilot testing: clinical experts without methodological training tended to over-trust AI outputs, whilst methodologists without clinical knowledge failed to catch domain-specific AI errors. Finally, transparency in reporting remains challenging for hybrid approaches. Determining exactly which aspects of the final analysis reflect AI outputs, human verification, or machine-assisted analysis requires careful documentation throughout the analytical process. We recommend maintaining detailed audit trails specifying which responses received human review, what changes were made to AI outputs, and the rationale for human overrides of machine classifications.

### When to use machine-assisted analysis

Our findings suggest specific contexts where machine-assisted analysis offers greatest value versus situations requiring primarily human analysis:

**Ideal contexts for machine-assisted analysis:**

- Large datasets (>500–1000 responses where setup overhead is justified)
- Responses with explicit theme mentions and clear sentiment expressions
- Time-critical research requiring rapid preliminary findings
- Preliminary exploration of unfamiliar datasets to identify key patterns
- Multi-site studies requiring standardised coding approaches
- Datasets requiring demographic segmentation or keyword frequency analysis

**When NOT appropriate:**

- Small samples (<500 responses) where setup time exceeds efficiency gains
- Highly sensitive topics requiring nuanced human judgement (e.g., suicide risk, abuse disclosures)
- Fully inductive research where categories must emerge organically from data
- Vulnerable populations where misclassification could cause harm
- Exploratory phenomenological studies prioritising depth over breadth
- Research requiring deep contextual interpretation of metaphor or narrative

**Hybrid approach recommended for:**

- Complex healthcare data containing clinical terminology and abbreviations
- Mixed methods studies requiring integration of qualitative and quantitative elements
- Policy research requiring both efficiency and interpretive nuance
- Studies where some themes are predefined whilst others may emerge

The decision should consider available resources, timeline, data sensitivity, and whether the research prioritises breadth (favouring machine assistance) or depth (favouring human analysis).

### Ethical and data governance considerations

Implementing machine-assisted analysis in healthcare research requires careful attention to consent, data protection, and ethical oversight.

Participants must be explicitly informed that AI tools will process their data, with clear explanation of what this entails. Our approach included several key safeguards: Ethics approval included specific consent language: “Your responses may be analysed using artificial intelligence tools to identify themes and patterns. These tools will not store your data permanently or use it for training purposes.” We provided transparency about which AI tools were used (Caplena, Infranodus), data retention periods (6 months post-project), and geographical processing locations (EU-only).

One of the most pressing concerns in the deployment of AI in healthcare is the potential for misclassification, where the system either fails to detect critical safety signals or misjudges the severity of a patient’s condition. This can occur due to limitations in the training data, oversimplified decision rules, or lack of contextual understanding [25].

In terms of GDPR Compliance, participants retained rights to opt-out of automated processing, request data portability, and request erasure. We implemented a tiered consent model allowing participants to consent to human analysis whilst opting out of AI processing if preferred. Regarding security measures, all data processing occurred within EU borders with no international transfers. Data was processed on UCL secure servers using encrypted transfer protocols (HTTPS/TLS 1.3). Access controls limited data viewing to named researchers, with comprehensive audit trails tracking all access and modifications. In terms of sensitive data protocols **- e**nhanced review protocols were implemented for responses potentially containing mental health content or safeguarding concerns. Any responses flagged by AI as potentially concerning underwent mandatory human review within 24 hours, with escalation pathways to the study’s clinical lead if needed.

Where commercial tool considerations were concerned, both Caplena and Infranodus confirmed data would not be used for model training and would be deleted after the specified retention period. We obtained written data processing agreements specifying these terms before uploading any participant data. Researchers considering machine-assisted analysis should consult their institutional data protection officer and ethics committee early in study design to ensure appropriate safeguards are implemented.

It is essential to highlight that while the previous NAP7 paper [12] thoroughly demonstrated the impact of peri-operative cardiac arrest on anaesthetic staff, the present study focuses on the methods used to analyse the co-related themes, sentiments, and experiences of anaesthetic staff working in peri-operative cardiac arrest management, and a comparison of human and machine-assisted analysis approaches. The co-related themes identified in our analysis, such as teamwork, communication, leadership, patient outcomes, and the debriefing process (Tables 1 and Tables 7-9), all played key roles in understanding the extent and nature of the impact of peri-operative cardiac arrest on anaesthetic staff. By focusing on these themes and their interrelationships, our study complements the findings of the previous NAP7 paper and provides a more nuanced understanding of staff experiences and the factors that shape them. Having established the methodological capabilities and limitations of machine-assisted Big Qual analysis, we now turn to the substantive findings generated by applying these methods to the NAP7 dataset. These findings demonstrate that hybrid approaches can produce clinically meaningful insights whilst significantly reducing analysis time [26–28].

Having established the methodological capabilities and limitations of machine-assisted Big Qual analysis, we now turn to the substantive findings generated by applying these methods to the NAP7 dataset. These findings demonstrate that hybrid approaches can produce clinically meaningful insights whilst significantly reducing analysis time. It is essential to highlight that whilst the previous NAP7 paper thoroughly demonstrated the impact of perioperative cardiac arrest on anaesthetic staff, the present study focuses on the methods used to analyse the co-related themes, sentiments, and experiences. The co-related themes identified in our analysis—teamwork, communication, leadership, patient outcomes, guidelines, debriefing, and wellbeing support (Tables 1 and Tables 7-9)—all played key roles in understanding the extent and nature of impact on anaesthetic staff.

### Alignment with patient safety theory

These themes align with established patient safety frameworks. The Swiss Cheese model [29] emphasises multi-faceted incident causation, whilst human factors research [30] highlights the importance of team dynamics and communication. Sujan and colleagues [26] focus on bridging the gap between work as imagined and work as done. Our findings about effective teamwork, communication, and leadership in successful cardiac arrest management support these theoretical frameworks, demonstrating that machine-assisted analysis can efficiently identify patterns aligned with established patient safety theory. This provides validation that hybrid analytical approaches can generate theoretically-grounded insights comparable to traditional human-only analysis, but with substantially reduced resource requirements.

Analysis revealed that effective teamwork, communication, and leadership were crucial factors in determining success of perioperative cardiac arrest management. Where teams worked well together with good communication, anaesthetists expressed more positive sentiments even when outcomes were suboptimal—ambiguous comments often contained mixed positive and negative elements. This underscores the importance of training and simulation exercises enhancing non-technical skills in high-stress situations [31,32]. Regarding guidelines, ambiguous responses highlighted challenges and need for targeted interventions. Comments such as “The QRH is useful but doesn’t go through the nuances and most likely causes for specific patient cohorts” emphasised the need for guidance on complex, patient-specific scenarios.

Debriefing analysis revealed positive, negative, and ambiguous sentiments. Whilst some respondents valued opportunities to discuss events, others reported negative experiences including criticism or inadequate training of debrief facilitators. Ambiguous responses like “It felt disorganised with no communication or debrief but I felt satisfied because all necessary steps were followed” highlight the need for balance between protocol adherence and open communication, emphasising importance of well-structured, psychologically safe debriefing processes.

The identification of the ‘ambiguous’ sentiment category has important clinical implications. These mixed-sentiment responses reveal critical insights about organisational resilience—situations where clinical teams succeeded despite system failures. For example, responses like “good outcome but poor leadership” identify specific quality improvement targets whilst acknowledging existing strengths. This nuanced understanding enables targeted interventions: rather than wholesale system replacements, organisations can build on what works whilst addressing specific weaknesses.

Policy implications are significant: the 38% of responses containing ambiguous sentiments suggest that binary ‘good/bad’ quality metrics miss crucial complexity in healthcare delivery. Responses such as “effective resuscitation but no debrief” point to specific gaps (post-event support) whilst confirming clinical competence. This granularity helps design interventions that address actual rather than perceived problems. Recognition of emotional complexity in responses like “relieved but guilty” or “proud but exhausted” highlights the need for support systems that acknowledge the full spectrum of healthcare professionals’ experiences rather than assuming simple positive or negative reactions to clinical events.

Psychosocial impact analysis encompassed diverse sentiments, with many ambiguous experiences. Negative experiences included lack of organisational support and increased anxiety in future similar cases. Positive experiences, though less common, highlighted importance of robust psychological support services and informal colleague support. These insights emphasise need for structured support systems including psychological services, peer support networks, and formal debriefing processes.

### Limitations

Our study has limitations that should be considered when interpreting the findings.

From a methodological perspective, our evaluation framework has certain limitations. The human-only analysis served as the gold standard against which we measured machine performance, which assumes perfect human analysis. However, as evidenced by our inter-annotator agreement of 0.82, human analysis itself has variability. In addition to this, while our detailed performance metrics (precision, recall, and F1-scores) provide a comprehensive evaluation, they may not fully capture the nuanced quality differences between human and machine analyses, particularly for complex qualitative data.

Additionally, we were unable to re-run the machine-assisted analysis in Caplena with the ‘ambiguous’ category included for direct comparison, as the platform does not offer functionality to add a fourth sentiment category beyond the standard positive, negative, and neutral classifications. While we could re-run the analysis to visualise blended sentiments (where statements display a mix of positive, negative, or neutral sentiment indicated by colour-coded bars), the technical constraints of the tool prevented us from fully integrating our human-identified ‘ambiguous’ category into the sentiment analysis model. This limitation highlights the current constraints of commercial AI tools in accommodating domain-specific or nuanced classification schemes that emerge during qualitative analysis. Additionally, the generalisability of our specific performance metrics (accuracy, precision, recall) may vary across different types of qualitative data. Whilst the methodology itself is transferable—indeed, Caplena and similar tools are designed for diverse text types including social media and interview transcripts—the efficiency gains and accuracy levels we report are specific to the structured survey responses in NAP7. Unstructured narratives may present different challenges, such as longer text segments, more complex narrative structures, or greater contextual dependency, which could affect both processing time and classification accuracy. Future research should establish performance benchmarks across various qualitative data types to better understand how data characteristics influence machine-assisted analysis outcomes.

Despite our adherence to information governance and data anonymisation, there are potential challenges and ethical considerations associated with implementing AI-assisted qualitative analysis in healthcare settings. Previous studies have highlighted the need for human oversight and interpretation [11] which we have tried to mitigate by introducing human input at each stage. However, the possibility of algorithmic bias in the initial training data of the AI computer tools used still perpetuates AI health inequalities based on lack of diversity in that specific AI training data. This bias often stems from a lack of diversity in the datasets used to train these algorithms, datasets that may disproportionately reflect the health profiles, behaviours, and outcomes of majority groups while neglecting the nuances and needs of marginalised communities. Without deliberate efforts to ensure inclusivity and equity in training data, these tools risk reinforcing systemic inequalities rather than mitigating them [33,34]. Additionally, the transformer-based models and machine-assisted analysis approaches employed in this study may have inherent biases in how they process language related to medical contexts, potentially affecting the thematic categorisation and sentiment analysis.

Furthermore, although we had five researchers from different backgrounds and specialties involved in the thematic analysis and three during the sentiment analysis process, we only had two researchers in the discussions of the percentage agreement of the human analysis. These researchers will have brought their own subjectivity to the analysis. If there had been more team members, the percentage agreement may have been lower as there may have been more varied results across multiple researchers. Another potential limitation of our study is the involvement of the same human analysts in both the human-only and machine-assisted analyses. As the analysts gained familiarity with the data during the human-only analysis, there is a possibility of learning or spillover of knowledge when conducting the machine-assisted analysis. This familiarity could have influenced their interpretations and decisions during the machine-assisted analysis, potentially leading to a more refined or biased understanding of the data compared to analysts without prior exposure. However, the use of a structured sentiment analysis framework (Fig 1) and the involvement of three analysts from different backgrounds and specialties (EB, EK, SM1) at times, aimed to mitigate this potential bias by ensuring consistency and reducing individual subjectivity in the analysis process. The initial investment in setting up, training, and fine-tuning the machine learning models represents a fixed overhead that becomes more cost-effective with larger datasets.

Regarding the response rate of the survey, while the overall response rate of 71% is similar to that of the NAP6 baseline survey [28], the response rates for each question progressively decreased from 100% to 92%, potentially introducing bias if non-respondents had different experiences or perspectives. However, the bias will be limited due to the whole population of anaesthetists being sampled, not just a representative sample within this population. Second, there is a risk of self-selection bias, as those with particularly memorable or impactful experiences might have been more inclined to participate, potentially skewing the results towards more extreme cases. Additionally, the specific focus on peri-operative cardiac arrests and the sole inclusion of anaesthetists’ perspectives may limit the generalisability of our findings to other healthcare settings, patient safety incidents, and the viewpoints of other resuscitation team members.

Despite these limitations, our study represents the analysis of the largest survey of anaesthetists to date examining individual preparedness, management, and experiences of peri-operative cardiac arrest. The use of Big Qual methods and an adaptive sentiment analysis and thematic analysis framework highlights the potential for applying these techniques to other areas of patient safety research. The insights gained can guide the development of tailored strategies and quality improvement initiatives to enhance patient safety.

### Future directions

Our findings highlight several avenues for advancing machine-assisted qualitative analysis in healthcare research. The limitations we identified in current commercial tools, particularly their inability to accommodate emergent categories like our ‘ambiguous’ sentiment classification, suggest that newer large language models (LLMs) may offer greater flexibility. Recent developments in generative AI, including GPT-4, Claude, and open-source alternatives, present opportunities to overcome the constraints we encountered. These models can potentially be prompted to identify emergent categories, handle ambiguous classifications, and better interpret clinical context through few-shot learning approaches. Future research should evaluate whether these capabilities translate into practical improvements for healthcare qualitative analysis. Key areas for development include: 1) fine-tuning of GPT-4 on our validated NAP7 dataset to create domain-specific model for anaesthesia research, 2) a comparative study to test GPT-4, Claude 3, and Gemini on identical 1,000-response subset to benchmark performance differences, 3) develop prompt engineering library optimised for healthcare qualitative data, test zero-shot vs few-shot learning approaches, 4) create open-source Python package allowing researchers to implement validated prompts without coding expertise 5) Collaborating with a university AI lab for technical support and apply for external funding to support the programme. As these technologies evolve, maintaining focus on augmenting rather than replacing human expertise will be essential for preserving the interpretive richness that defines qualitative inquiry.

## Conclusion

This study adds to the literature as one of a few systematic comparisons of human-only versus machine-assisted analysis for Big Qual data in healthcare research, establishing benchmark metrics for efficiency gains and accuracy trade-offs. Using the NAP7 baseline survey as a test case, we demonstrate that machine-assisted analysis can reduce analysis time by over 60% whilst maintaining acceptable accuracy levels for many research purposes.

**Key findings**

- Efficiency Gain: Machine-assisted analysis reduced total analysis time by 64.3% (from 255 hours to 88 hours), with the greatest efficiency improvement in theme identification (98.6% reduction from 210 hours to 3 hours).
- Accuracy Trade-off: Machine-assisted approach achieved 78% thematic analysis accuracy and 72% sentiment classification accuracy compared to the human gold standard, demonstrating an acceptable trade-off for the substantial efficiency gains.
- Processing Capacity: The machine-assisted approach increased processing capacity from ~20 responses/hour to ~57 responses/hour, representing a 185% improvement in throughput that enables analysis of larger datasets.
- Sentiment Distribution: Machine analysis initially classified 36% of responses as neutral, whereas human analysis identified only 7% as truly neutral with another 7% categorised as ambiguous, highlighting the importance of human oversight for nuanced interpretations.
- Category Performance: Performance varied across sentiment and thematic categories, with F1-scores ranging from 0.60 (neutral sentiment) to 0.84 (patient outcome theme), indicating machine learning performed better on more clearly defined categories.
- Human-Machine Agreement: Inter-annotator agreement between human annotators (κ = 0.82) exceeded human-machine agreement (κ = 0.72), indicating good but not perfect alignment between approaches.
- Complementary Strengths: Human expertise proved essential for identifying the “ambiguous” sentiment category and refining thematic classifications, while machine efficiency dramatically reduced the time required for initial processing.

Our comprehensive evaluation using multiple performance metrics demonstrated that the machine-assisted analysis significantly streamlined the process of analysing the large dataset, enabling the efficient identification of key themes and sentiments.

The insights gained from this study also complement the findings of the previous NAP7 paper [12] by providing a more in-depth understanding of the Big Qual methodology employed to investigate staff experiences.

Our findings have important implications for qualitative health research methodology. First, the hybrid approach combining AI efficiency with human interpretive expertise represents a sustainable methodology for handling Big Qual datasets that would otherwise be impractical to analyse comprehensively. Second, current commercial AI platforms’ inability to accommodate emergent analytical categories constrains their utility for fully inductive qualitative research, highlighting the need for AI tools designed specifically for qualitative research paradigms. Third, our results demonstrate that whilst AI can identify patterns aligned with pre-defined frameworks, human analysts remain essential for recognising novel insights and contextual nuances—supporting arguments for ‘augmented’ rather than ‘automated’ qualitative analysis.

This work establishes a foundation for further methodological development in machine-assisted qualitative analysis. Priority areas include developing AI tools that can adapt to emergent coding schemes, establishing optimal machine-assisted models for different research paradigms, and creating quality standards specific to machine-assisted qualitative research. As we continue to refine these hybrid approaches, interdisciplinary collaboration between domain experts, qualitative methodologists, and AI specialists will be essential to ensure reliability, validity, and ethical application.

The evolution of Big Qual research in healthcare will likely involve increasingly sophisticated integration of human and machine capabilities. By establishing clear methodological benchmarks and acknowledging both the potential and limitations of current tools, this study contributes to building a robust framework for future machine-assisted qualitative research that maintains the depth and rigour expected of qualitative inquiry whilst enabling analysis at unprecedented scales.

## Supporting information

S1 Appendix Collaborator list. (DOCX)

S2 Appendix NAP7 baseline survey. (PDF)

S3 Appendix Flowchart showing individual anaesthetists’ survey and question responses. (TIFF)

S4 Appendix Performance metrics definitions. (TIFF)

S5 Appendix Sentiment analysis formula. (TIFF)
