# Supplementary material for: Comparing human vs. machine-assisted analysis to develop a new approach for Big Qualitative Data Analysis
Source: PLOS Digit Health. 2026 Feb 25;5(2):e0000576. doi: 10.1371/journal.pdig.0000576 (PMC12935260; doi:10.1371/journal.pdig.0000576)
Supplement: S2 Appendix — (PDF) [file pdig.0000576.s002.pdf]

Baseline survey of all anaesthetists

1. Welcome to the NAP7 Baseline Survey: Anaesthetists and Anaesthesia Associates

Many thanks for taking part in this first phase of NAP7: Perioperative Cardiac Arrest in the UK.

This survey will take 5-10 minutes to complete. Please complete in one sitting, otherwise your data may be lost.

NAP7 will start on 16th June 2021. Please report cases of perioperative cardiac arrest in adults and children to your Local Coordinator.

2. Welcome to the NAP7 Baseline Survey: Anaesthetists and Anaesthesia Associates

Some questions viewed through NHS browsers may not appear correctly. You may wish to use your own device.

SCOPE: This survey is about perioperative cardiac arrest including your attitudes and experience, training and demographics.

METHODS: This survey is for all anaesthetists and all anaesthesia associates working in all UK hospitals (NHS and Independent sector). All grades, including trainees, should complete this survey.

ALL responses are confidential and anonymous.

3. Knowledge, training and attitudes of perioperative cardiac arrest

* 1. When was your **most recent** training in advanced life support including chest compressions and defibrillation? Tick one option for each row.

Resuscitation Council UK (RCUK) or equivalent courses: *e.g. ALS, ILS, APLS, EPALS*

In-house hands-on training sessions: departmental or hospital

	Instruct at least yearly	Within 1 - 2 years	Within 2 - 4 years	> 4 years	Not applicable (e.g. do not treat children)	None	Can't recall
RCUK adult or equivalent	◯	◯	◯	◯	◯	◯	◯
In-house adult 'hands-on training'	◯	◯	◯	◯	◯	◯	◯
RCUK paediatric or equivalent	◯	◯	◯	◯	◯	◯	◯
In-house paediatric 'hands-on training'	◯	◯	◯	◯	◯	◯	◯

* 2. Do you agree or disagree with the following statement?

I am confident in leading the management of cardiac arrest on the operating table.

Strongly agree	Agree	Neither agree or disagree	Disagree	Strongly disagree
○	○	○	○	○

Please provide reason(s) for your answer.

* 3. Do you agree or disagree with the following statements?

	Strongly agree	Agree	Neither agree or disagree	Disagree	Strongly disagree
I have received sufficient training in the management of **intraoperative** cardiac arrest.	○	○	○	○	○
I would benefit from more training in the management of **intraoperative** cardiac arrest.	○	○	○	○	○
I am confident in leading a debrief process.	○	○	○	○	○
I would benefit from training in how to conduct a debrief.	○	○	○	○	○
I am confident in leading communication with relatives/next of kin after an intraoperative cardiac arrest.	○	○	○	○	○

* 4. Do you agree or disagree with the following statement?

Existing guidelines for the management of perioperative cardiac arrest are sufficient.

Strongly agree	Agree	Neither agree or disagree	Disagree	Strongly disagree
○	○	○	○	○

Please provide reason(s) for your answer.

* 5. In your opinion, what are the **three most common** causes of perioperative cardiac arrest?

State 'not sure' if you are unsure.

Cause 1

Cause 2

Cause 3

* 6. In an anaesthetised 50-year old ASA 2 patient, without an arterial line, who developed hypotension, **whilst treating causes of profound hypotension**, what would you use as an indication to start chest compressions? Tick all that apply.

(for BP, please choose the highest BP option at which you would start chest compressions)

- [ ] Systolic BP 51 – 60 mmHg
- [ ] Systolic BP 41 – 50 mmHg
- [ ] Systolic BP 31 – 40 mmHg
- [ ] Systolic BP ≤ 30 mmHg
- [ ] Unrecordable BP
- [ ] No palpable peripheral pulse
- [ ] No palpable central pulse
- [ ] Very low end-tidal CO_2
- [ ] None of these
- [ ] I'm not sure
- [ ] Other (please specify)

* 7. What indications would you use to start chest compressions for the previous question (Q6) if the patient was aged 75, hypertensive and ASA 3? Tick all that apply.

(for BP, please choose the highest BP option at which you would start chest compressions)

- [ ] Systolic BP 51 – 60 mmHg
- [ ] Systolic BP 41 – 50 mmHg
- [ ] Systolic BP 31 – 40 mmHg
- [ ] Systolic BP ≤ 30mmHg
- [ ] Unrecordable BP
- [ ] No peripheral pulse
- [ ] No central pulse
- [ ] Very low end tidal CO_2
- [ ] None of these
- [ ] I'm not sure
- [ ] Other (please specify)

4. Personal experience of perioperative cardiac arrest

The following questions are about your recent experience (within the last 2 years).

Cardiac arrest is defined as the need for at least 5 chest compressions and/or defibrillation in a patient having a procedure under the care of an anaesthetist.

This question is for arrests occurring between your first hands-on contact with the patient at the start of anaesthesia care until handover to another clinician (e.g. leaving recovery area to the ward, handover to ICU).

PLEASE EXCLUDE cases where:
1. defibrillation is a planned, normal, or expected part of the procedure (e.g. during VT ablation);
2. chest/internal cardiac compressions and/or defibrillation occur during cardiopulmonary bypass;
3. patients in whom chest compressions and/or defibrillation were not started when cardiac arrest occurred;
4. patients who received <5 chest compressions.

* 8. Within the **last 2 years**, how many cases of perioperative cardiac arrest do you recall being involved with (**present during or managed**)?

- ◯ 0
- ◯ 1
- ◯ 2
- ◯ 3
- ◯ 4
- ◯ 5
- ◯ More than 5
- ◯ Can't recall

5. Most recent experience of perioperative cardiac arrest

The following questions are about your most recent perioperative cardiac arrest in which you were involved (present during or managed).

* 9. What was the location of the perioperative cardiac arrest?

* 10. What PPE precautions did you use during the management of the perioperative cardiac arrest?

Airborne = FFP3, fluid repellent long sleeved gown, gloves, eye protection
Droplet = Fluid resistant surgical mask, apron, gloves +/- eye protection
Contact = Standard face mask, apron, gloves +/- eye protection
Other = Standard face mask +/- gloves or no PPE

	Airborne precautions	Droplet precautions	Contact precautions	Other	Can't recall
Just before/ at the time of arrest	◯	◯	◯	◯	◯
During the arrest	◯	◯	◯	◯	◯

* 11. How would you describe your experience in managing a perioperative cardiac arrest **in PPE** compared to before the COVID-19 pandemic?

Much worse	Worse	Neither better or worse	Better	Much better	Not applicable
◯	◯	◯	◯	◯	◯

Please provide reason(s) for your answer.

* 12. What was the approximate age of the patient?

- ≤1 years old
- >1 – 5 years old
- >5 – 12 years old
- >12 – 18 years old
- >18 – 65 years old
- >65 years old
- Age not known
- Can't recall
- Prefer not to say

6. Most recent experience of perioperative cardiac arrest

* 13. What was the most likely **‘suspected or confirmed’** primary cause of the perioperative cardiac arrest?

- Airway/breathing problem
- Cardiac/cardiovascular (including arrhythmia, MI, bleeding, sepsis, thromboembolic)
- Neurological problem
- Regional anaesthesia (including high neuraxial block, LA toxicity)
- Metabolic (including electrolyte disorder, and malignant hyperthermia)
- Anaphylaxis
- Error, drug or equipment problem
- Uncertain cause - multiple comorbidities and/or extreme age or frailty
- Unknown cause
- Can't recall

7. Most recent experience of perioperative cardiac arrest

* 14. What was the most likely **‘suspected or confirmed’** primary cause of the perioperative cardiac arrest?

Choose single best option.

If option not available, press 'previous' below for another category OR choose 'other' and state below.

- Failed mask ventilation
- Failed supraglottic airway placement
- Failed intubation
- Laryngospasm
- Cannot intubate cannot oxygenate (CICO)
- Unrecognised oesophageal intubation
- Airway haemorrhage
- Regurgitation
- Aspiration of gastric contents
- Aspiration of blood
- Severe hypoxaemia
- Bronchospasm
- High airway pressure / obstructive ventilation
- Gas trapping / high iPEEP
- Hypercapnia
- Hypocapnia
- Pneumothorax
- Tension pneumothorax
- Endobronchial intubation
- Pulmonary embolism
- Ventilator disconnection
- Wrong gas supplied/unintentional connection to air
- Other (please specify)

8. Most recent experience of perioperative cardiac arrest

* 15. What was the most likely **PRIMARY** cause of the perioperative cardiac arrest?

Choose single best option.

If option not available, press 'previous' below for another category OR choose 'other' below.

- Major haemorrhage
- Bradyarrhythmia
- Tachyarrhythmia
- Isolated severe hypotension (central vasopressors considered/started)
- DC cardioversion
- Cardiac ischaemia
- Cardiac tamponade
- New AF
- Ventricular tachycardia
- Ventricular fibrillation
- Complete heart block
- Pulmonary embolism
- Fat embolism
- Bone cement implantation syndrome
- Amniotic fluid embolism
- Air embolism
- CO_2 embolism
- Septic shock
- Anaphylaxis
- Local anaesthetic toxicity (excessive dose and/or wrong route)
- Incompatible blood transfusion
- Addisonian crisis
- Vagal outflow – e.g. pneumoperitoneum, oculo-cardiac reflex
- High neuraxial block
- Other (please specify)

9. Most recent experience of perioperative cardiac arrest

* 16. What was the most likely **PRIMARY** cause of the perioperative cardiac arrest?

Choose single best option.

If option not available, press 'previous' below for another category OR choose 'other' below.

- ◯ Intracranial haemorrhage (including subarachnoid haemorrhage)
- ◯ Raised intracranial pressure (e.g. new fixed/dilated pupil(s))
- ◯ Seizure
- ◯ Vagal outflow – e.g. pneumoperitoneum, oculo-cardiac reflex
- ◯ High neuraxial block
- ◯ Cushing’s Response/Coning
- ◯ Neurogenic/spinal shock
- ◯ Stroke
- ◯ Local anaesthetic toxicity (excessive dose and/or wrong route)
- ◯ Other (please specify)

10. Most recent experience of perioperative cardiac arrest

* 17. What was the most likely **PRIMARY** cause of the perioperative cardiac arrest?

Choose single best option.

If option not available, press 'previous' below for another category OR choose 'other' below.

- ◯ High neuraxial block
- ◯ Inadvertent neuraxial block during regional block
- ◯ Local anaesthetic toxicity (excessive dose and/or wrong route)
- ◯ Other (please specify)

11. Most recent experience of perioperative cardiac arrest

* 18. What was the most likely **PRIMARY** cause of the perioperative cardiac arrest?

Choose single best option.

If option not available, press 'previous' below for another category OR choose 'other' below.

- New significant acidosis/acidaemia
- Significant hyperkalaemia
- Significant hypokalaemia
- Significant hypermagnesaemia
- Significant hypomagnesaemia
- Significant hyperthermia
- Significant hypothermia
- Malignant hyperthermia
- Addisonian crisis
- Other (please specify)

12. Most recent experience of perioperative cardiac arrest

* 19. What was the most likely **PRIMARY** cause of the perioperative cardiac arrest?

Choose single best option.

If option not available, press 'previous' below for another category OR choose 'other' below.

- Drug error
- Incompatible blood transfusion
- High neuraxial block
- Inadvertent neuraxial block during regional block
- Local anaesthetic toxicity (excessive dose and/or wrong route)
- Malignant hyperthermia
- Wrong gas supplied/unintentional connection to air
- Equipment failure
- Equipment lack
- Ventilator disconnection
- Other (please specify)

13. Most recent experience of perioperative cardiac arrest

* 20. Did the patient survive the cardiac arrest?

ROSC = return of spontaneous circulation

- ◯ Died - no ROSC
- ◯ Died - transient ROSC (<20 min)
- ◯ Died - CPR stopped due to patient's known wishes (e.g. previous DNACPR decision or ReSPECT form)
- ◯ Survived cardiac arrest but died prior to hospital discharge
- ◯ Survived initial resuscitation and still in hospital
- ◯ Survived initial resuscitation but final outcome unknown
- ◯ Survived to hospital discharge
- ◯ Unknown

* 21. Were you present at the start of anaesthesia?

- ◯ Yes
- ◯ No
- ◯ Can't recall

* 22. Who was present in the room **at the start of anaesthesia**? Exclude yourself if you were present.

Tick all that apply.

- ☐ Consultant
- ☐ SAS doctor
- ☐ Post CCT or CESR doctor
- ☐ ST5+ or equivalent
- ☐ ST3-4 or equivalent
- ☐ CT2 or equivalent
- ☐ CT1 or equivalent – after Initial Assessment of Competence (IAC)
- ☐ CT1 or equivalent – before completion of Initial Assessment of Competence (IAC)
- ☐ Other (please specify)
- ☐ Anaesthesia Associate
- ☐ ODP/Anaesthetic nurse/Anaesthetic assistant
- ☐ Nurse/HCA e.g. scrub or recovery nurse
- ☐ Surgical team
- ☐ None
- ☐ Can’t recall
- ☐ Not applicable

* 23. List the number of anaesthesia providers (include yourself in the numbers) present **during the management** of the cardiac arrest?

	0	1	2	3	≥4	Can't recall
Consultant	◯	◯	◯	◯	◯	◯
SAS doctor	◯	◯	◯	◯	◯	◯
Post CCT or CESR doctor	◯	◯	◯	◯	◯	◯
ST5+ or equivalent	◯	◯	◯	◯	◯	◯
ST3-4 or equivalent	◯	◯	◯	◯	◯	◯
CT2 or equivalent	◯	◯	◯	◯	◯	◯
CT1 or equivalent – after Initial Assessment of Competence (IAC)	◯	◯	◯	◯	◯	◯
CT1 or equivalent – before completion of Initial Assessment of Competence (IAC)	◯	◯	◯	◯	◯	◯
Anaesthesia Associate	◯	◯	◯	◯	◯	◯
ODP/Anaesthetic nurse/Anaesthetic assistant	◯	◯	◯	◯	◯	◯

Other (please specify)

* 24. Who did you perceive to be leading the cardiac arrest?

* 25. Was a specific guideline used to assist in the management of perioperative cardiac arrest?

- ◯ Yes
- ◯ No
- ◯ Can't recall

14. Most recent experience of perioperative cardiac arrest

* 26. How was the specific guideline accessed? Tick all that apply.

- ☐ Smartphone
- ☐ Laminate
- ☐ In treatment pack
- ☐ Printed copy in theatre
- ☐ Other (please specify)
- ☐ Computer/tablet
- ☐ Memory
- ☐ Can't recall
- ☐ Not applicable

* 27. Was the theatre list or anaesthetic on-call shift stopped early?

- ◯ Yes – paused
- ◯ Yes – list stopped (includes cancelling remaining patients or transferring to care by a different team)
- ◯ No
- ◯ Not applicable (e.g. last case on list)
- ◯ Don't know
- ◯ Other (please specify)

* 28. Did any members of the team stand down from clinical activity* immediately after the event? Tick all that apply.

**does not include a break to document events or communicate with family, next of kin or other clinicians*

- ☐ Yes – I stood down
- ☐ Yes – some of the team
- ☐ Yes – all of the team
- ☐ No – because this was the end of the list or shift anyway
- ☐ No one stood down (e.g. continued with the next case)
- ☐ Can't recall

15. Most recent experience of perioperative cardiac arrest

* 29. How did you or the team members stand down from clinical activity* immediately after the event? Tick all those that best describe this.

**A break does not include a break to document events or communicate with family, next of kin or other clinicians.*

- ☐ Took a short break* (e.g. <1 hour)
- ☐ Took a sustained break* (e.g. >1 hour)
- ☐ Theatre list terminated early
- ☐ Anaesthetic on-call shift terminated early
- ☐ Can't recall
- ☐ Not applicable
- ☐ Other (please specify)

* 30. How satisfied or dissatisfied were you with the quality of the management of the arrest?

Very satisfied	Satisfied	Neither satisfied or dissatisfied	Dissatisfied	Very dissatisfied
○	○	○	○	○

Please provide reason(s) for your answer.

* 31. Who was the main member of the resuscitating team to directly communicate with the patient's relatives / next of kin following the event?

- ○ Me
- ○ Another member of the team
- ○ Not applicable (e.g. no next of kin immediately available)
- ○ Can't recall

16. Most recent experience of perioperative cardiac arrest.

* 32. Excluding yourself, who was the main team member to directly communicate with the patient's relatives/next of kin following the event? Tick all that apply.

- ☐ Consultant anaesthetist
- ☐ Trainee anaesthetist
- ☐ SAS anaesthetist
- ☐ Consultant surgeon
- ☐ Trainee surgeon
- ☐ SAS surgeon
- ☐ Consultant from ICU
- ☐ ICU Trainee
- ☐ SAS from ICU
- ☐ Anaesthesia associate
- ☐ Nursing staff
- ☐ Physician
- ☐ Don't know
- ☐ Not applicable
- ☐ Other (please specify)

* 33. Was there a debrief relating to the case?

- ◯ Yes – I attended
- ◯ Yes – unable to attend (work duties)
- ◯ Yes – unable to attend (on leave)
- ◯ Yes – I was not invited
- ◯ Yes – I decided not to attend
- ◯ No, but there will be
- ◯ No, none planned
- ◯ Don’t know
- ◯ Other (please specify)

17. Most recent experience of perioperative cardiac arrest

* 34. When did the debrief occur?

- ◯ Immediately after the event (hot debrief)
- ◯ Delayed period after the event (cold debrief)
- ◯ Both
- ◯ Can't recall
- ◯ Not applicable
- ◯ Other (please specify)

* 35. What type of debrief was carried out? Tick all that apply.

- ☐ Informal
- ☐ Formal (i.e. with a trained facilitator)
- ☐ One-to-one
- ☐ Group
- ☐ Other (please specify)
- ☐ Trauma risk management (TRiM)
- ☐ Critical Incident Stress Debriefing (CISD)
- ☐ Don't know
- ☐ Not applicable

* 36. Do you agree or disagree with the following statement?

I was satisfied with the debrief process following the event.

Strongly agree	Agree	Neither agree or disagree	Disagree	Strongly disagree	Not applicable
◯	◯	◯	◯	◯	◯

Please provide reason(s) for your answer.

* 37. How was the case (or will the case be) reviewed? Tick all that apply.

- ☐ Mortality and morbidity meeting
- ☐ Audit/ QI / governance meeting
- ☐ Internal investigation meeting e.g. root cause analysis
- ☐ Structured judgement/ mortality review
- ☐ Non-anaesthetic departmental meeting
- ☐ Other (please specify)
- ☐ Multi-specialty meeting, grand round or similar
- ☐ Serious incident framework
- ☐ No review has been performed or planned
- ☐ Don't know
- ☐ Not applicable

* 38. Was there an inquest or equivalent (e.g. Procurator Fiscal)?

- ◯ Yes
- ◯ Pending
- ◯ No
- ◯ Don't know
- ◯ Prefer not to say
- ◯ Not applicable

* 39. Was the case followed by legal proceedings?

- ◯ No
- ◯ Too early to know
- ◯ Yes
- ◯ Don’t know
- ◯ Prefer not to say
- ◯ Not applicable

* 40. Do you agree or disagree with the following statement?

I feel satisfied with the way in which the case was followed up and reviewed.

Strongly agree	Agree	Neither agree or disagree	Disagree	Strongly disagree
◯	◯	◯	◯	◯

Please provide reason(s) for your answer.

* 41. What type of support have you received for this most recent case of perioperative cardiac arrest?

	Yes	No	Prefer not to say	Not needed
Informal support (e.g. from colleagues)	◯	◯	◯	◯
Formal support from dedicated experienced senior anaesthetist	◯	◯	◯	◯
Formal hospital wellbeing support	◯	◯	◯	◯

Other (please specify)

* 42. Has the cardiac arrest episode impacted on your ability to deliver patient care?

- ◯ Yes
- ◯ No
- ◯ Not sure
- ◯ Prefer not to say

18. Most recent experience of perioperative cardiac arrest

* 43. How did the cardiac arrest episode impact your ability to deliver care?

19. Career experience of perioperative cardiac arrest

The following questions are about your CAREER EXPERIENCE of perioperative cardiac arrest.

* 44. In your career, have any of your experiences of perioperative cardiac arrest (as the primary anaesthetist and those attended as a helper) had an **ADVERSE** impact on your **professional/work** life?

- ◯ Yes
- ◯ No
- ◯ Prefer not to say
- ◯ Not sure
- ◯ Not applicable - no prior experience of perioperative cardiac arrest

20. Career experience of perioperative cardiac arrest

* 45. How did your experiences have an **adverse** impact on your **professional/work** life? Please tick all that apply.

- ☐ Time off work
- ☐ Loss of professional confidence
- ☐ Work related anxiety and stress
- ☐ Impacted relationship with colleagues
- ☐ Change in job plan
- ☐ Change hospital
- ☐ Change career
- ☐ Complaint about your performance
- ☐ Internal investigation about your performance
- ☐ Suspension
- ☐ GMC referral
- ☐ GMC investigation
- ☐ Civil Litigation
- ☐ Criminal investigation
- ☐ Prefer not to say
- ☐ Not applicable - no prior experience of perioperative cardiac arrest
- ☐ Other (please specify).

* 46. In your career, have any of your experiences of perioperative cardiac arrest (as the primary anaesthetist and those attended as a helper) had a **POSITIVE** impact on your **professional/work life**?

If yes, please provide details below.

- ◯ Yes
- ◯ No
- ◯ Not sure
- ◯ Prefer not to say
- ◯ Not applicable - no prior experience of perioperative cardiac arrest

If yes, please provide details

* 47. In your career, have any of your experiences of perioperative cardiac arrest (as the primary anaesthetist and those attended as a helper) had an **ADVERSE** impact on your **personal** life?

- ◯ Yes
- ◯ No
- ◯ Not sure
- ◯ Prefer not to say
- ◯ Not applicable - no prior experience of perioperative cardiac arrest

21. Career experience of perioperative cardiac arrest

* 48. How did your experiences have an **adverse** impact on your **personal** life? Please tick all that apply.

- ☐ Impacted relationship with partner and/or children
- ☐ Impacted relationship with relatives
- ☐ Impacted relationship with friends
- ☐ Needed medical advice or care
- ☐ Other (please specify)
- ☐ Needed psychological support
- ☐ Prefer not to say
- ☐ Not applicable - no prior experience of perioperative cardiac arrest

* 49. In your career, have any of your experiences of perioperative cardiac arrest (as the primary anaesthetist and those attended as a helper) had a **POSITIVE** impact on your **personal life**?

If yes, please provide details below.

- ◯ Yes
- ◯ No
- ◯ Not sure
- ◯ Prefer not to say
- ◯ Not applicable - no prior experience of perioperative cardiac arrest

If yes, please provide details

22. Demographics and workplace characteristics

The following questions are about your current work practices.

* 50. What gender do you identify yourself as?

- ◯ Female
- ◯ Male
- ◯ Other
- ◯ Prefer not to say

* 51. What is your age?

- ◯ <25 years old
- ◯ 25 – 35 years old
- ◯ 36 – 65 years old
- ◯ >65 years old
- ◯ Prefer not to say

* 52. What is your current role in Anaesthesia?

____

* 53. How long have you been an anaesthetist/anaesthesia associate? Please specify the number of years or months.

Years ____________________

Months ____________________

* 54. Do you currently work out of hours (weekend and/or nights)?

- ◯ Yes
- ◯ No

* 55. Do you have a subspecialty? Tick all that apply.

- [ ] Airway/ Head & neck
- [ ] Paediatrics
- [ ] Cardiothoracics
- [ ] Intensive care
- [ ] Neurosurgery
- [ ] Obstetrics
- [ ] Day case anaesthesia
- [ ] Orthopaedics
- [ ] Bariatrics
- [ ] Transplant
- [ ] Trauma
- [ ] Regional anaesthesia
- [ ] Vascular
- [ ] Eyes
- [ ] None of the above
- [ ] Not applicable
- [ ] Other (please specify)

* 56. What type of organisations do you currently work in?

- ( ) NHS
- ( ) Independent sector (non-NHS)
- ( ) Both

57. What country or region are you reporting from?

23. Thank you for your contribution to the NAP7 baseline survey.

Please contact your Local Coordinator that you have completed this survey.
You will then receive a certificate for completing the NAP7 Baseline Survey.

NAP7 starts on Wednesday 16th June 2021.

Please inform your Local Coordinator of all cases which may fulfil the criteria of perioperative cardiac arrest.

For more details please visit https://www.nationalauditprojects.org.uk/NAP7-Home#pt
