# Supplementary figures and images for: Comparing human vs. machine-assisted analysis to develop a new approach for Big Qualitative Data Analysis
Source: PLOS Digit Health. 2026 Feb 25;5(2):e0000576. doi: 10.1371/journal.pdig.0000576 (PMC12935260; doi:10.1371/journal.pdig.0000576)

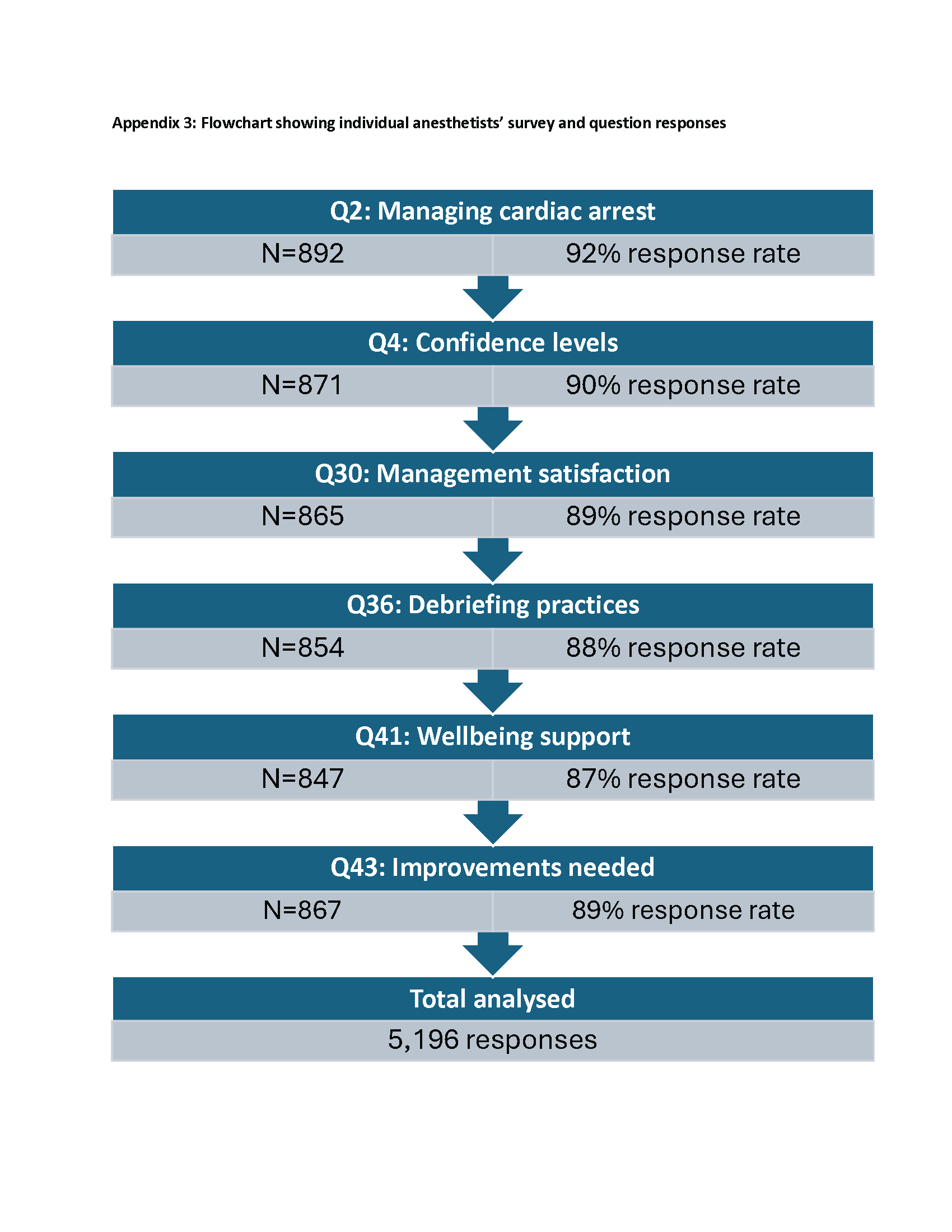

Supplement: S3 Appendix — (TIFF) [file pdig.0000576.s003.tiff]

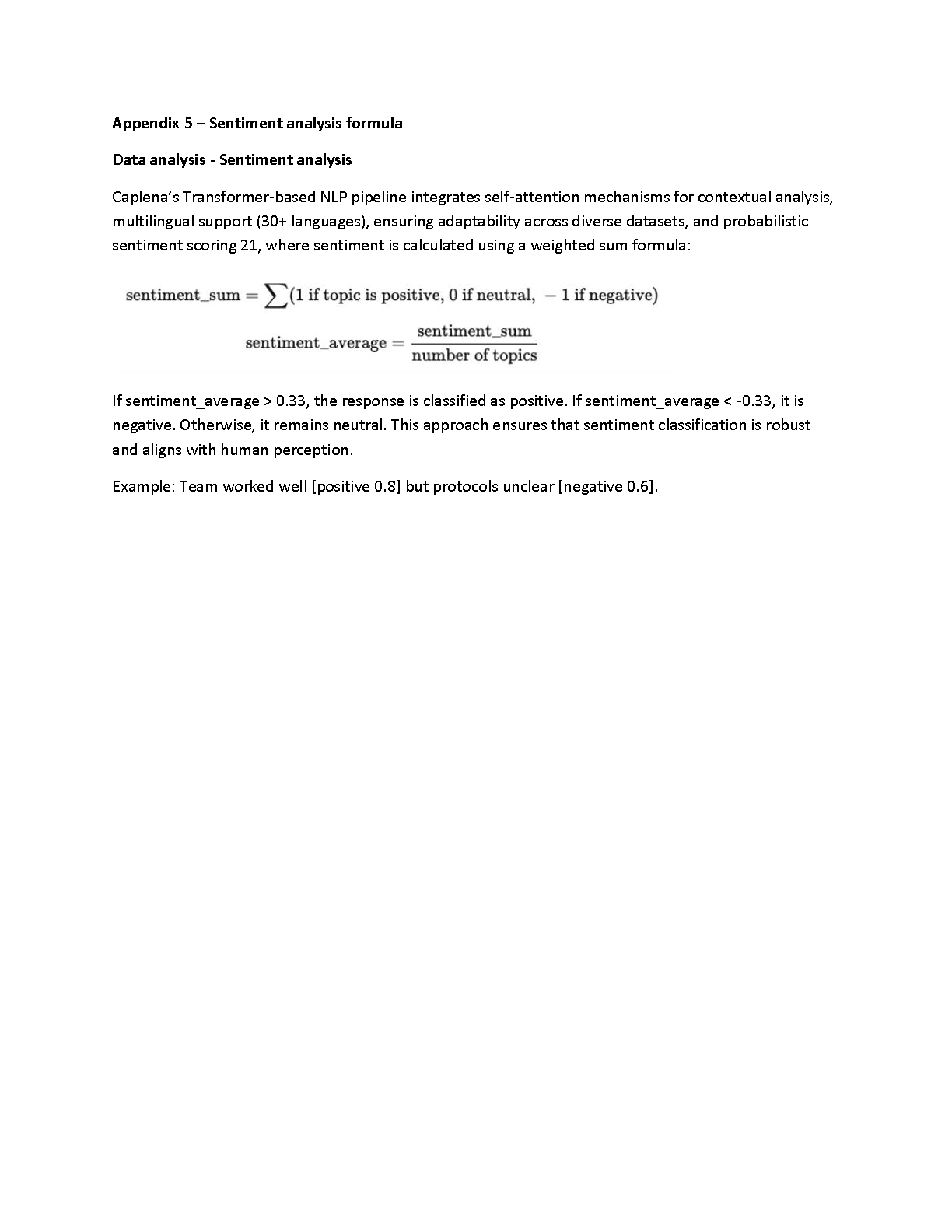

Supplement: S5 Appendix — (TIFF) [file pdig.0000576.s005.tiff]
